# Deep learning of cuneiform sign detection with weak supervision using transliteration alignment

**DOI:** 10.1371/journal.pone.0243039

**Published:** 2020-12-16

**Authors:** Tobias Dencker, Pablo Klinkisch, Stefan M. Maul, Björn Ommer

**Affiliations:** 1 Heidelberg Collaboratory for Image Processing, Interdisciplinary Center for Scientific Computing, Heidelberg University, Heidelberg, Germany; 2 Department of the Languages and Cultures of the Near East, Institute for Assyriology, Heidelberg University, Heidelberg, Germany; University of Central Florida (UCF), UNITED STATES

## Abstract

The cuneiform script provides a glimpse into our ancient history. However, reading age-old clay tablets is time-consuming and requires years of training. To simplify this process, we propose a deep-learning based sign detector that locates and classifies cuneiform signs in images of clay tablets. Deep learning requires large amounts of training data in the form of bounding boxes around cuneiform signs, which are not readily available and costly to obtain in the case of cuneiform script. To tackle this problem, we make use of existing transliterations, a sign-by-sign representation of the tablet content in Latin script. Since these do not provide sign localization, we propose a weakly supervised approach: We align tablet images with their corresponding transliterations to localize the transliterated signs in the tablet image, before using these localized signs in place of annotations to re-train the sign detector. A better sign detector in turn boosts the quality of the alignments. We combine these steps in an iterative process that enables training a cuneiform sign detector from transliterations only. While our method works weakly supervised, a small number of annotations further boost the performance of the cuneiform sign detector which we evaluate on a large collection of clay tablets from the Neo-Assyrian period. To enable experts to directly apply the sign detector in their study of cuneiform texts, we additionally provide a web application for the analysis of clay tablets with a trained cuneiform sign detector.

## Introduction

Used in most of the Ancient Near East for a period of over three thousand years until the beginning of the Current Era, the cuneiform script is the oldest known writing system in the world, providing us with invaluable records of early human history across all spheres of life.

Most cuneiform texts were written on palm-sized clay tablets by impressing an angular stylus that left a wedge-shaped mark. Groups of wedges (*cunei* in Latin, hence the name cuneiform) formed *cuneiform signs*, of which several hundred have been documented in cuneiform sign lists [1]. In contrast to script written on papyrus or paper, clay tablets are more resilient to the passing of time and so far over 500’000 such tablets have been found. Only a third of the discovered tablets have been thoroughly studied and many more remain in the ground to be recovered in the future.

Even for experts, the process of reading and analyzing clay tablets is difficult and time-consuming [2–4]. In an initial reading, an Assyriologist first identifies groups of wedges as individual cuneiform signs and determines for each identified sign its *sign code class*, a classification according to a common sign list. To better understand the cuneiform text and update the initial reading accordingly, the Assyriologist then iterates between two steps combining epigraphic and linguistic findings: First, in an *autograph* the cuneiform signs are mapped from 3D to a 2D drawing in a wedge-by-wedge manner. Second, in a *transliteration* for each cuneiform sign one of several possible readings (in the original language) is recorded in Latin script. Due to the special design of the transliteration system, it is possible to retrieve the original sign code class of each sign from its reading. If we mention transliterations in the remainder of this article, we will refer to this sign-by-sign representation in terms of sign code classes rather than readings. In the last step of the analysis, a translation of the readings is created.

**Fig 1:**
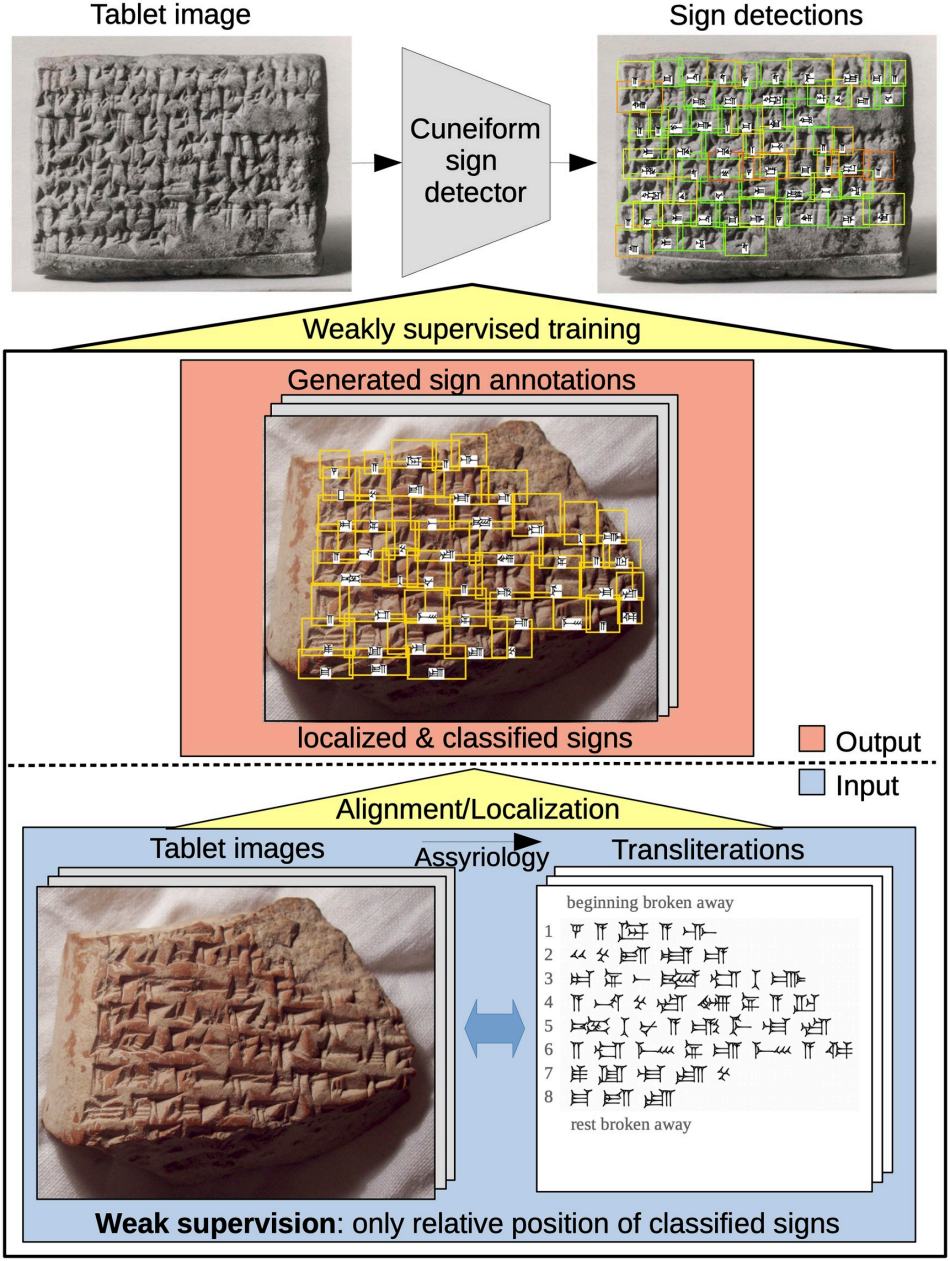
Overview of our approach. To support Assyriologists we train a cuneiform sign detector to localize and classify cuneiform signs in tablet images. The sign annotations necessary for training the sign detector are automatically generated by localizing signs of existing transliterations in their tablet images. This alignment turns weak supervision of the transliteration into full supervision in terms of bounding boxes. Image material at the top is shared by The Metropolitan Museum of Art under a CC0 license. Image material below by the authors.

To support Assyriologists in their analysis, we want to facilitate the decipherment of cuneiform script. In particular, our goal is to obtain a *cuneiform sign detector* that outputs for a tablet image where a sign is located (bounding box enclosing the sign) and what sign code class it belongs to (according to Borger’s sign list [1]) as shown in Fig 1. Our aim is not to replace Assyriologists by automatically generating a transliteration or translation, but rather to support them with sign detection as a fundamental part of reading cuneiform script. For this purpose, we also present a web application that makes the cuneiform sign detector readily available to Assyriologists, which we illustrate in S1 Video.

**Fig 2:**
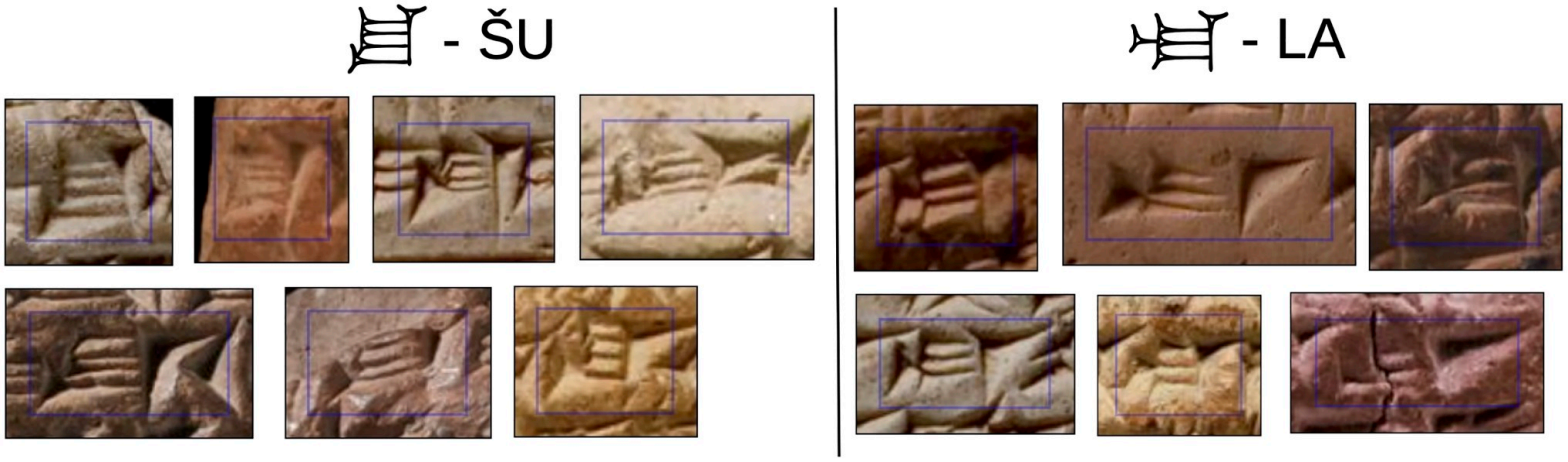
The problem of fine-grained sign similarity. Detecting individual signs on a cuneiform tablet usually requires the expertise of an Assyriologist. Cuneiform signs of different sign code classes may look very similar, while signs of the same sign code class may look very different. Image material by the authors.

While in many writing systems the recognition of individual characters is not an issue, detecting cuneiform signs is difficult for several reasons. In many scripts whitespace between characters simplifies detection significantly, because it allows us to separate localization and classification in two consecutive steps. However, cuneiform signs are often inscribed without space between neighboring signs making their localization and classification interdependent [5]. Sign similarity of over 900 different signs increases the difficulty further as shown for two sign code classes in Fig 2. Since most cuneiform signs are constructed from very few types of wedges (in Neo-Assyrian: lying, standing and diagonal) that are combined in various spatial configurations, different sign code classes are often very similar (high inter-class similarity). Meanwhile, cuneiform signs of the same sign code class can be very dissimilar in their appearance if written by different scribes (high intra-class variance). Many tablets have been damaged over the course of time and thus complicate sign detection further.

Cuneiform tablets have been digitized in various forms that strongly differ in their availability and production cost. Cuneiform tablets are best studied in their original form as 3D objects: signs are often found not only on front and back but also along edges, and their visibility depends on lighting and viewpoint. Thus working on 3D scans of tablets is desirable [6–10]. From 3D scans, 2D representations are derived that are used for recognition [11, 12]. Similarly, autographs can be used as input for a detector [13–16]. However, the creation of 3D scans or autographs is expensive and time-consuming. In contrast, we rely on 2D images of tablets (composite image of different tablet sides) which are readily available online [17].

There are three categories of computer vision approaches for text or handwriting recognition: Character-based [18–23], word-based [24–27], or line-based approaches [28–30]. Unlike the character-based approach that localizes and classifies a sign in one step, the word-based and line-based approaches only localize the words or lines of a text, which are then transcribed into a sequence of signs without explicit sign-level localization. In the context of cuneiform script, line-based approaches suffer from noise introduced by the additional line detection step, since lines are often damaged and tricky to follow due to gaps and offsets. A word-level approach is ill-suited due to polyvalence of cuneiform signs that allows to assemble a word from various often ambiguous combinations of signs. We adopt a character-based approach that results in a sign detector that directly outputs bounding boxes for individual cuneiform signs. Sign-level bounding boxes help to explain the decision making of the detector and thus provide a crucial asset for Assyriologists.

Ideally, we would follow a standard machine-learning approach and train a sign detector from thousands of annotated examples of cuneiform signs (supervised learning) [19–21, 25]. However, a sufficient amount of training data in the form of bounding box annotations is not available and its collection is time-consuming and requires expert knowledge. Moreover, due to the changes in cuneiform script over time, we would need to collect multiple training sets. Instead of manually creating new annotations, it is more natural to use existing transliterations that are essential part of Assyriologist’s work and available for thousands of tablets via the ORACC websites [31].

While the transliteration provides a sign-by-sign representation of the tablet content, it does not explicitly localize each sign in the 2D tablet image. If we want to learn how to detect cuneiform signs from tablet images and corresponding transliterations (weakly supervised learning), we need to localize the transliterated signs for each 2D image, i.e. solving the inverse problem with respect to sign detection as shown in Fig 1. We can understand the transliteration as a Rosetta stone [32, 33] for learning how to detect cuneiform script: By aligning image and transliteration, we identify correspondences in different representations of the same underlying text. These correspondences provide examples for training a better sign detector that in turn improves the alignment of image and transliteration. Repeating this process across hundreds of tablet images in parallel enables our approach to gradually learn how to detect signs starting with easy to locate, often occurring signs and step-by-step filling in the remainder. Instead of teaching the algorithm by annotating thousands of examples manually, it learns from existing 2D tablet images and corresponding transliterations.

From a machine-learning perspective, the transliteration provides *weak supervision*, in contrast to the fully supervised setting with available bounding box annotations. Character-based methods [22, 23] have been proposed that use word-level annotations in a weakly-supervised learning framework. While they infer the positions of individual characters from the bounding box around a word, our approach localizes cuneiform signs by aligning the image of a clay tablet with its transliteration which usually involves multiple lines of cuneiform script. Additionally, our method works without an initial set of training annotations. Beyond weak supervision, there are unsupervised approaches. Using synthetic data is a common approach for (pre-)training a detector for typed texts unlike handwriting [24, 27]. Recently, a method for the generation of cuneiform script has been proposed with promising results [34], but the outputs still lack the diversity of real-world data needed to train a robust sign detector. [35] uses a probabilistic model to infer the most likely transcription of historic printed texts. [36] proposes a generative adversarial network to train a character classifier from unpaired data of line segmentations and unaligned text transcriptions, only requiring them to be remotely related. While these approaches show promising results for printed English text, they are not directly applicable to non-Latin handwritten scripts with far more characters that are written without regular spacing between words and that possess a high degree of self-similarity.

Weakly supervised learning is an important research area as it is one of the most common learning scenarios found in the wild. Learning to detect cuneiform script challenges us to find new ways to bridge the gap between weak and full supervision, enabling state-of-the-art cuneiform sign detection for over a hundred sign code classes without requiring manual annotations. While our proposed solution deals with cuneiform sign detection, similar problems (fine-grained detection, few annotations) occur in other ancient scripts that might profit from our weakly supervised approach.

Our main contributions are summarized as follows: (i) Since reading cuneiform script is difficult and time-consuming even for experts, we propose to train a deep neural network-based sign detector that can localize and classify cuneiform signs directly in 2D images of clay tablets. To this end, (ii) this article describes a novel method to supervise the sign detector training with weak supervision provided by existing transliterations—instead of requiring extra manual annotation of ten thousands of cuneiform signs. (iii) To the best of our knowledge, we are the first to demonstrate a cuneiform sign detector that performs well on a large and diverse dataset and detects over a hundred different sign code classes. (iv) We release the first large-scale dataset for the challenging problem of cuneiform sign detection in 2D images with several thousand signs annotated by Assyriologists. (v) The approach is made available with code, web application and trained models, and it is applicable to cuneiform scripts of different periods paving the way for large-scale digital analysis in the field of Assyriology.

In the following we describe our approach in detail, evaluate our approach and its components thoroughly in a number of experiments, and finish with a discussion of our approach and its implications for computer vision and Assyriology.

## Approach

Training a cuneiform sign detector requires thousands of sign annotations. Instead of requiring manual annotations, our weakly supervised approach generates sign annotations by automatically aligning hundreds of tablet images with their transliterations in parallel. We propose a *iterative learning* procedure that alternates between generating sign annotations and training the sign detector. We model the sign detector as a convolutional neural network (CNN) whose representation of cuneiform signs is updated in each iteration of iterative training.

**Fig 3:**
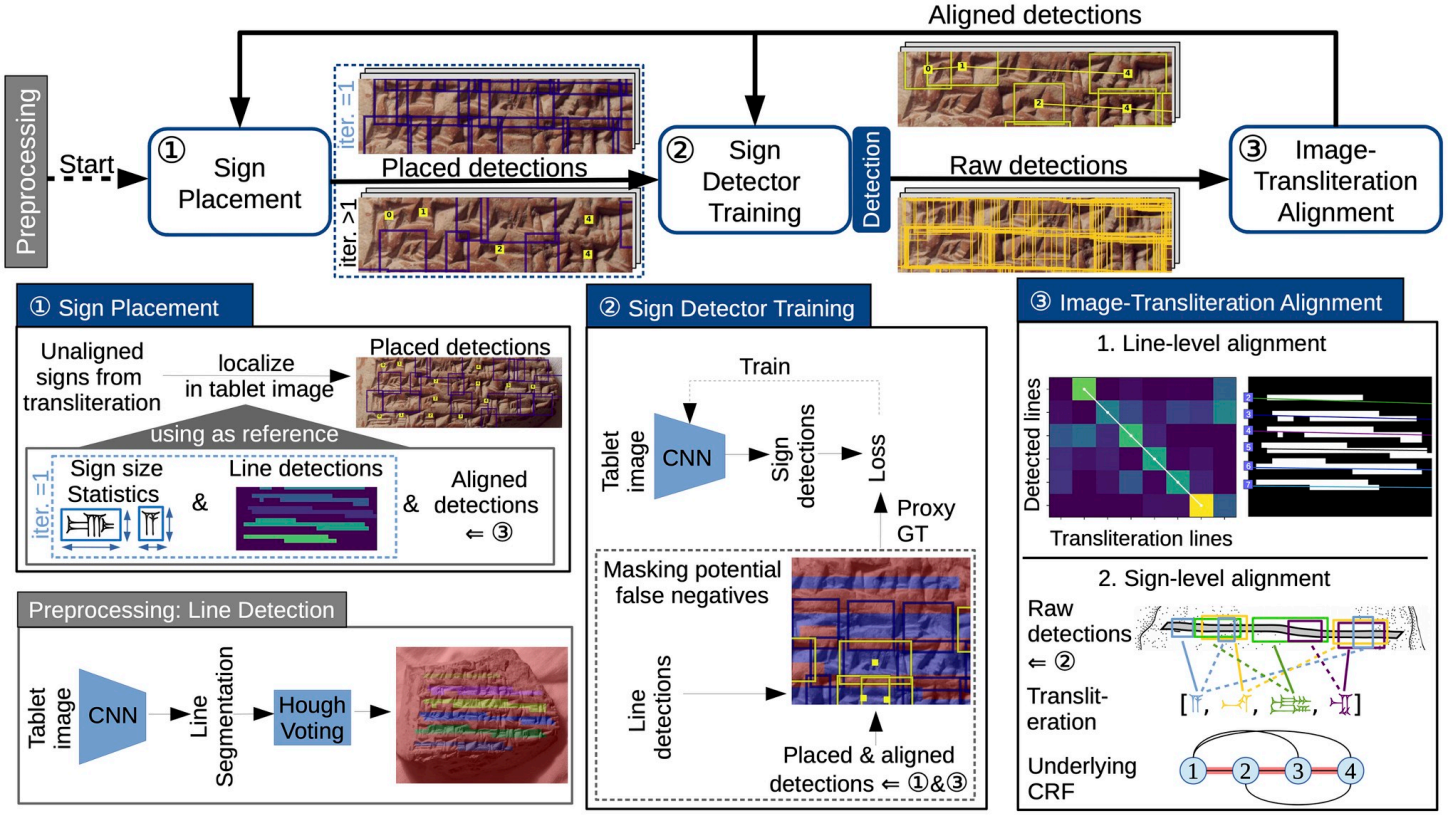
Weakly supervised training of sign detector comprises an iterative training loop with three steps. In each iteration the sign placement method localizes transliterated signs in the tablet image using detected lines and aligned detections as reference points. In the second step, the aligned & placed detections serve as sign annotations for training a sign detector. In the third step, the image-transliteration alignment filters the raw sign detections produced by the sign detector after the second step. Only the raw detections that are consistent with the transliteration and the line geometry are selected. The resulting aligned detections are very reliable sign annotations, which boost sign detector training in the next iteration. As pre-processing, we detect lines in all tablet images to simplify localization and alignment. Image material by the authors.

To start iterative training, an initial set of sign annotations is required to train a first sign detector. Since the transliteration only provides the relative positions of cuneiform signs, we propose a sign placement method that relies on line detections to turn the relative positions of the transliterated signs into actual bounding boxes in the tablet image (*placed detections*). To keep improving the sign detector, it is necessary to increase the quality and quantity of generated sign annotations from iteration to iteration. When applying the trained sign detector on tablet images (of the training set), this produces *raw detections* with few true positive (TP) and many false positive (FP) sign detections. By aligning transliterated signs with raw detections in a tablet image, our method identifies a reliable subset of raw detections (*aligned detections*) that are most likely TPs and can serve as sign annotations for the next training iteration. When aligned detections become available after the first iteration, the task of sign placement shifts to localizing the unaligned signs left in the gaps between aligned detections. As summarized in Fig 3, our approach performs three key steps in each iteration of iterative learning:

1. Localize transliterated signs in the tablet image using detected lines and aligned detections as reference points (*sign placement*).
2. Train a sign detector using the generated aligned & placed detections as sign annotations (*sign detector training*).
3. After applying the sign detector, refine the raw detections by optimizing the alignment between transliteration and tablet image (*image-transliteration alignment*).

To better understand why the placement and alignment methods are essential for iterative learning, we can think about their interaction in terms of exploration vs. exploitation: The placement method provides an initial sign localization and later fills the gaps between aligned detections which include valuable training samples of sign code classes yet to be learned by the detector (exploration). In contrast, the alignment method converts weak supervision from transliterations into high-quality aligned detections (exploitation). The ability to explore new sign code classes is of particular importance due to the problem of sign code class imbalance: Many sign code classes are rare in cuneiform script because their occurrence follows a discrete Pareto distribution as shown in Fig B in S1 File. During iterative learning, the sign detector is biased to perform best on sign code classes with the most training data, while ignoring rare sign code classes. To counter this effect, sign placement injects sign annotations that are difficult-to-learn or belong to rare sign code classes into the iterative learning process.

### Line detection

Before iterative learning, line detection is performed as a pre-processing step on all tablet images. Cuneiform text is commonly inscribed along lines. Since these lines are easy to detect, we can simplify the alignment between tablet image and transliteration. Given the lines in a tablet image, the full alignment problem is decomposed into a line-level alignment (that matches detected with transliterated lines) and for each line a sign-level alignment (that matches detected with transliterated signs). Knowing the position of each line can effectively reduce sign localization in a 2D tablet image to an 1D search along a line, significantly constraining the number of possible solutions.

We propose a line detection method for cuneiform script that benefits the three steps of our learning method described in the following paragraphs. As shown in Fig 3, it is implemented as a CNN for line segmentation (pixel-wise labeling of line/no line) whose output is post-processed using a straight line Hough transform [37] voting for line detections. We provide implementation details of the line segmentation network, its training, and the post-processing in Sect. A2 of S1 File.

### Sign placement

Transliterations only provide a relative placement of some signs to another, but not the exact locations of individual cuneiform signs in the tablet images (bounding boxes), essential for training a sign detector. In the first iteration of iterative training, the sign placement method combines the relative sign positions from the transliteration with line detections in the tablet image in order to generate an initial set of sign annotations as shown in Fig 3. The detected lines serve as reference lines along which to place sign bounding boxes. For the line-level alignment, we match the detected lines with transliterated lines from top to bottom. The heights of sign bounding boxes are derived from the distance between detected lines. The widths are computed by multiplying the sign heights with a class-specific sign width from a pre-computed statistic. Even though only a small percentage of placed sign detections are correct, applying this method to hundreds of tablet images still yields a substantial number of valid sign annotations that our learning method can exploit iteratively.

In later iterations of iterative learning, the sign placement additionally leverages the aligned sign detections in order to localize the remaining unaligned signs from the transliteration. The aligned detections serve as additional reference points throughout the tablet image that complement the reference provided by the detected lines. In particular, the localization of signs close to aligned detections is improved. Therefore, the more aligned detections become available during iterative training, the better the remaining unaligned signs can be localized. Also the line-level alignment is now adopted from the previous image-transliteration alignment step. More details on the implementation of sign placement are in Sect. A3 of S1 File.

Besides providing an initialization for our learning method, in later iterations the sign placement step drives the learning of sign code classes not yet learned by the sign detector. Placed detections correspond to transliterated signs that have either not been detected or not been aligned successfully, and thus have the potential to be valuable training data for the next round of detector training. Through iterative learning with placed detections, the sign detector gradually learns to distinguish new sign code classes.

### Sign detector architecture

The CNN of the sign detector is based on the Single Shot Multi-Box Detector (SSD) architecture [38] for generic object detection, which we adapt for the detection of cuneiform signs. It defines a dense grid of anchor boxes over the input image and outputs for each box a class prediction as well as a regression of box coordinates for a better fit. It uses a class-generic bounding box regression, which is beneficial for classes with few examples [39]. We also extend the sign detector with a feature pyramid network [40] that enables SSD to cover additional image scales to detect signs of different sizes. This increases the sign detector’s robustness against scale changes in the cuneiform script. More implementation details are provided in the Sect. A4 of S1 File.

As backbone architecture of the sign detector, we use a MobileNet-v02 [41] with the width multiplier set to 0.625, since it shows good performance and has few learnable parameters which helps with regularization. We further reduce its capacity by removing the last inverted block and increasing the stride of the first layer to two for faster downsampling. A comparison of other backbone architectures is provided in the Sect. C5 of S1 File.

### Sign detector training

When we train a cuneiform sign detector, we improve its internal representation of cuneiform signs. Different cuneiform signs share the same representation that captures the commonalities between signs. Since cuneiform signs are all constructed from very similar parts (wedges), success in the distinction of some sign code classes already lays the foundation for the distinction of yet unlearned sign code classes. Erroneous and ambiguous training samples are difficult to integrate into the learned representation and tend to be ignored. Thus the sign detector training provides in itself an critical regularization that filters out the noise in the generated sign annotations.

In each iteration of iterative training, the sign detector is trained on the aligned & placed detections as shown in Fig 3. The training of a sign detector heavily relies on difficult negative examples of sign detections (hard negatives), e.g. bounding boxes centered between two lines. During sign detector training, hard negatives are automatically sampled in the close proximity of the generated sign annotations (positives). However, the generated aligned & placed detections only approximate ground truth annotations, and misplaced detections as well as undetected signs are part of the iterative learning process. Since aligned & placed detections only account for a subset of all visible signs in the tablet images, the automatic sampling of hard negatives can erroneously produce positive sign detections labeled as negatives (false negatives) which inhibits detector training. Our proposed method allows detector training despite incomplete and sometimes erroneous sign annotations. While we do not know the ground truth sign locations of all visible signs, we consider the line segmentation as a reliable indicator for their presence. By means of the line segmentation, we mask and discard all potential FP training samples in regions of the tablet image that are not covered by the aligned & placed detections (blue regions outside of bounding boxes in Fig 3). The resulting proxy ground truth (GT) incorporates all generated sign annotations and many valuable hard negatives, while reducing the negative impact of false negatives.

Our approach allows the integration of expert knowledge in the form of manual annotations, e.g. correct some of the generated sign detections. While our learning approach works weakly supervised (without full supervision), we find that complementing the weak supervision (i.e. transliterations) with a small number of sign annotations is beneficial in such a fine-grained detection setting. For this purpose, we extend the sign detector training with an optional fine-tuning step that trains the sign detector with a reduced learning rate on training samples annotated by experts. This form of training is referred to as *semi-supervised* learning because sign annotations are only available for a small subset of the training data. The rest of the training data remains weakly supervised. During our experiments, we evaluate the sign detector training in both cases, purely weakly supervised and semi-supervised. In the Sect. A4 & A7 of S1 File we provide details on false-positive masking, fine-tuning, and hyperparameters for training.

### Image-transliteration alignment

There are many causes like damaged signs or wedge-shaped cracks that can fool a cuneiform sign detector to produce FP sign detections. We close the iterative training loop of our approach by including an image-transliteration alignment step that serves as a filter for the raw detections of the sign detector. By selecting only sign detections that are successfully aligned with transliterated signs, we obtain a set of reliable detections for the next iteration of iterative training.

By extracting reliable aligned detections from the raw output of the sign detector, the image-transliteration alignment addresses two problems caused by an inherent discrepancy between tablet image and its transliteration: First, the transliteration does not record every visual detail found in the tablet image like writing style, gaps in the lines, damage to the clay tablet. Second, the transliteration often goes beyond what is actually visible in a tablet image. An Assyriologist may infer additional cuneiform signs due to context knowledge like multiple text sources, language understanding etc. While broken and damaged parts of the script are often documented, the level of detail varies between transliterations.

The optimization of the image-transliteration alignment yields a set of aligned detections that is consistent with the top-down information from transliteration and line geometry as well as the bottom-up information from line and sign detections. Due to line detection, we can decompose the problem of aligning transliterated signs of the transliteration with raw detections in the tablet image into two individual alignment problems as shown in Fig 3: 1) Aligning transliteration lines with detected lines in the tablet image (line-level alignment). 2) Solving the line-wise alignment problem of localizing signs from the transliteration in the detected line (sign-level alignment). Compared to a full-image alignment, this line-wise decomposition results in local sub-problems with constrained search spaces that increase the chance of finding a correct solution and speed up computation. Additionally, difficult lines (sub-problems) can be skipped without impacting easier lines that can already be aligned in early iterations of our learning method. In the following we formulate both line-level and sign-level alignment as individual optimization problems:

The line-level alignment is modeled as a path search problem through the matrix of all possible alignments where the shortest path represents the best line alignment (see in Fig 3 upper half of alignment box). Details on the implementation of the line-level can be found in Sect. A5 of S1 File.

The sign-level alignment is formulated as a pictorial structures model [42] where the best sign alignment is found by minimizing the energy of a conditional random field model (CRF). A sign alignment assigns each sign in the transliteration exactly one out of many available raw detections of the sign detector (see in Fig 3 lower half of alignment box). The energy of a sign alignment is determined by the energy functions (potentials) of the CRF that are designed according to our prior knowledge of what constitutes a reliable sign alignment (e.g. correct order of signs, relative straight line).

To construct a CRF for a cuneiform line (as shown in Fig 3 lower half of alignment box), we create a node for each sign in the transliteration vector and add edges to form a fully-connected graph. Nodes and edges of the CRF are associated with unary and pairwise potentials that rate the local appearance of signs as well as their joint spatial configuration. In the case of unary potentials, we rely on the confidence of the raw detection and the vertical offset of the detection bounding box from the detected line. In the case of pairwise potentials, we consider the horizontal distance between signs, the overlap of their bounding boxes, and the angle between the line drawn through the center points of two signs and the detected line.

By optimizing for an assignments associated with a low energy, the raw detections are selected that best match our prior knowledge. In the case that no matching raw detections can be assigned to a transliterated sign, the outlier label is assigned that incurs as fixed energy penalty. Thus the sign-level alignment still produces useful results, even if a subset of a line cannot be assigned. Details on the implementation of the sign-level alignment, including the formal definition of the energy functions, can be found in Sect. A6 of S1 File.

## Results

Our experiments have the goal to study the individual components of our learning approach as well as the full iterative training of a cuneiform sign detector on a large scale. For this purpose, we introduce a large dataset for cuneiform sign detection with clay tablets and corresponding transliterations. A small subset of the clay tablets has been manually annotated with bounding boxes to enable a quantitative performance analysis during testing.

### Datasets and evaluation metrics

We concentrate on the Neo-Assyrian stage of cuneiform writing (c. 900 BCE—600 BCE), due to the number of texts readily available and the standardized form of the cuneiform writing by that time. The dataset of clay tablet images with their transliterations mostly originates from two different sources:

- Transliterations of Neo-Assyrian tablets found in the *State Archives of Assyria online* (SAAo) [31, 43], as digitized by the ORACC project [44].
- Clay-tablet images made available through the *Cuneiform Digital Library Initiative* (cdli) [17].

For the Neo-Assyrian period, the SAAo series are invaluable, delivering a diverse corpus of texts, including royal correspondence, divination, and literary texts or international treaties, among other genres. We distilled our dataset by first extracting the usable transliterations from SAAo and then collating them with the available images in cdli. Only tablets with an available transliteration and image were included in our corpus. Clay tablets were often inscribed on both sides and even on the edges. Thus images in cdli are composites of different views (inscribed sides of a clay tablet). We segment those composite images in order to separate the different views into individual images and then associate the segmented views with their corresponding transliteration.

Table 1 provides an overview of our collected dataset. For training, the number of available segmented views (images) depends on the form of training (i.e. weakly supervised, or semi-supervised): For purely weakly supervised training, we use 2983 segmented views with their associated transliterations (Train-TL). For semi-supervised training, we additionally use up to 67 views (Train-BB) where all visible signs are annotated with bounding boxes (BB). For validation, we use 31 annotated views from Train-BB as a hold-out validation set (with 1753 sign annotations) to optimize hyperparameters. For testing, we use another 57 annotated views (Test).

**Table 1:** Composition of the two train sets and the test set.

Set	BB annotations	Signs	Views	Tablets	Weakly sup.	Semi- sup.
Train-TL	✗	185399	2983	1745	✓	✓
Train-BB	✓	4663	67	47	✗	✓
Test	✓	3446	57	34	-	-

Out of the c. 900 different cuneiform sign code classes catalogued in Borger’s sign list [1], we restricted ourselves to a subset of 186 sign code classes that are present in the Train-BB and Test set. This subset encompasses the most frequent sign code classes found in SAAo and represents the majority of the classes used during the Neo-Assyrian period. In Figs B & C of S1 File we plot the frequency distribution of cuneiform sign code classes and visualize them as font signs to provide an impression of their variability.

For evaluation, a sign detection is considered a TP, if its bounding box and a ground truth box overlap more than 50% (intersection-over-union) and their labels match, otherwise it is considered an FP. The quality of the generated sign detections (aligned & placed detections) are evaluated in terms of precision, recall and F2-score, each of which is averaged across the 186 sign code classes. We emphasize the recall of the generated sign annotations since exploring examples, that are difficult-to-learn or belong to rare sign code classes, is essential during iterative training, and even more so due to the natural sign code class imbalance in the dataset. The performance of the sign detector on an individual sign code class is measured in terms of average precision (AP) which is the standard metric for object detection [45]. The overall sign detector performance is measured in terms of mean AP (mAP), the average of the AP values of all 186 sign code class.

### Effect of alignment and placement method

In our first experiment, we analyze the impact of the alignment and placement methods on the quality of sign detections. For this purpose, we track the change in precision, recall and F2-score from raw detections to aligned detections and eventually to the union of aligned & placed detections. The experiment is conducted on the test set, starting from the raw detections of a sign detector obtained after three rounds of iterative training. Then the alignment and placement methods are applied consecutively.

**Fig 4:**
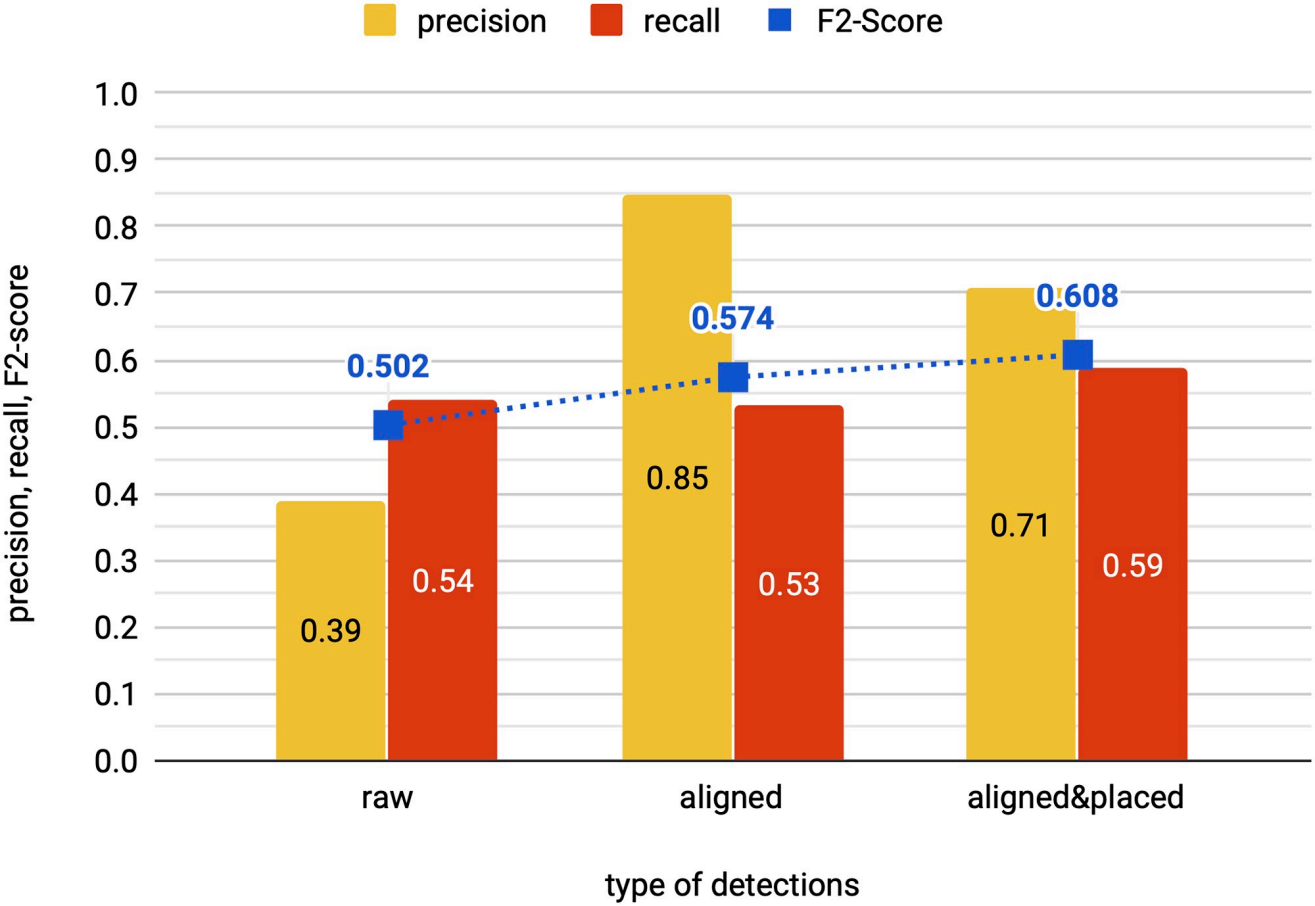
Effect of alignment and placement method on quality of detections. Our alignment method leverages weak supervision from transliterations to boost precision of aligned detections (aligned) compared to raw detections (raw). The placement method further adds detections (placed) that are combined with aligned detections. We report the change in precision, recall and F2-score of the detections produced by our method on the test set.

In Fig 4 we compare the quality of raw detections to aligned detections and to the union of aligned & placed detections. Using the weak supervision from transliterations helps to increase precision by aligning TP detections while discarding FPs. The alignment method produces aligned detections with a significantly increased precision (reduction in FPs) and a recall very close to the one of raw detections (while maintaining TPs). Since the alignment method effectively serves as a filter for the raw detections, the recall of aligned detections cannot surpass the recall of raw detections. In contrast, the placement method increases the recall of aligned & placed detections by filling the gaps between aligned detections with unaligned signs from the transliteration. While the placement method increases the recall of aligned & placed detections, it also leads to a decrease in precision. We find that our deep learning method is robust to the small loss in precision and the increase of recall helps with learning difficult and unexplored classes over the course of iterative training.

### Effect of iterative training

**Fig 5:**
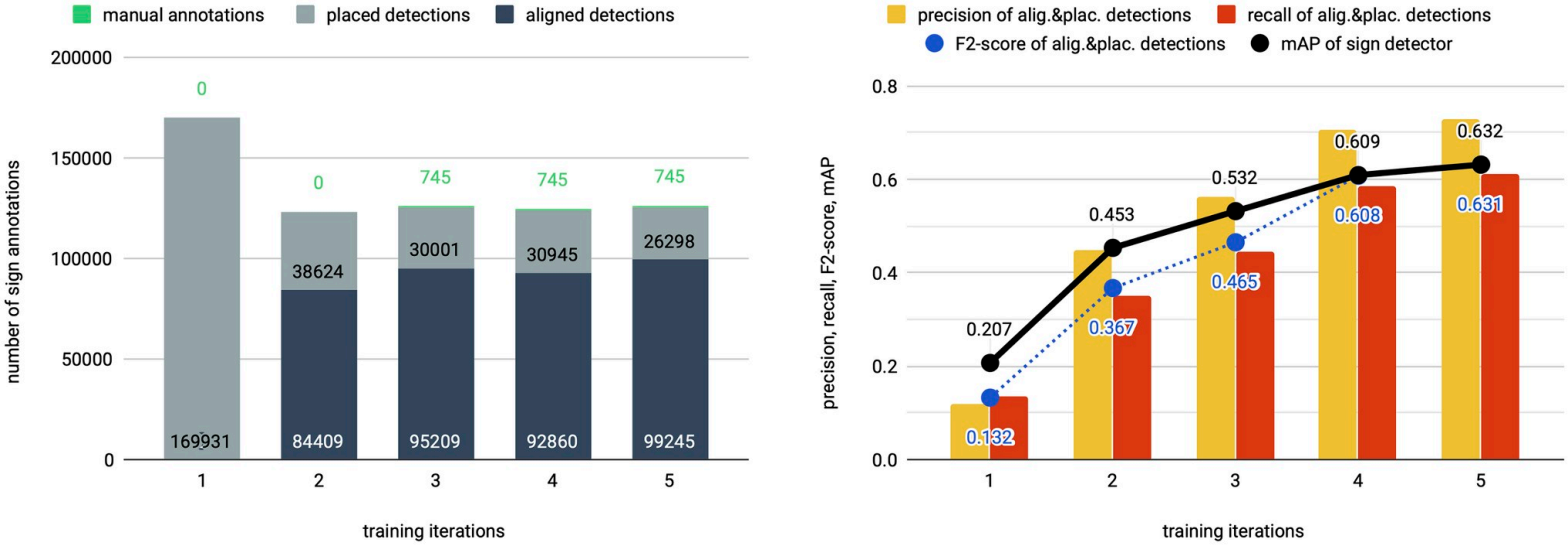
Effect of iterative training on ratio of generated detection types and detection performance. The first two iterations (1–2) are weakly supervised (w/o manual annotations) followed by three semi-supervised iterations (3–5) which include fine-tuning on 745 manual sign annotations. (a) The ratio of detection types varies between iterations. The training starts with only placed detections which are replaced with an increasing number of aligned detections over the course of training. (b) The precision and recall of the generated sign annotations and the performance (mAP) of the sign detector on the test set. To improve the sign detector, the quality and quantity of generated sign annotations needs to increase over the course of training.

Our second experiment investigates how iterative training gradually improves the sign detector in order to eventually detect all signs in a tablet image. To understand the effect of iterative training, we report for each iteration the composition of the aligned & placed detections in Fig 5, their F2-score as well as the performance of the sign detector after training in terms of mAP in Fig 5. Iterative training naturally suffers from a phenomenon called *drift* [46], that refers to performance degradation of the sign detector due to small errors in the aligned & placed detections that build up over the course of training. If the drift effect outweighs the performance gain between iterations, we stop iterative training.

The first and second training iterations of this experiment are weakly supervised without any manual sign annotations. In the first iteration, only placed detections are available for detector training of which less than twelve percent are TPs when evaluated on the test set. Despite the low precision of the placed detections, the sign detector training produces a first detector with 20.7 mAP. By aligning more than 80k detections, our weakly supervised approach increases the sign detector performance significantly (45.3 mAP) in the second iteration. An additional third iteration of purely weakly supervised training fails to improve performance. In particular, we find that sign localization of aligned & placed detections worsens due to poorly placed bounding boxes and this drift cancels out the positive effects of iterative training (see also Fig F of S1 File). On the third iteration, we add 745 manual sign annotations from Train-BB to the more than 100k already generated annotations (aligned & placed detections) as shown in Fig 5. This corresponds to the semi-supervised case of iterative training that entails fine-tuning the sign detector on a small number of manual annotations during each iteration. By semi-supervised learning, the localization drift is reduced and the performance increases up to 63.2 mAP. On the fourth iteration of semi-supervised training, we find no performance improvement and thus stop iterative training. We again observe that further improvement is inhibited by drift that causes small errors to accumulate, and can neither be corrected by the alignment nor by the fine-tuning step.

In weakly supervised as well as in semi-supervised training, the performance gain is strongly linked to the gradual improvement of aligned & placed detections as measured by the F2-score. At the end of semi-supervised training, over sixty percent of the transliterated signs in train set B are correctly grounded in the tablet images, providing over 100k training samples with an F2-score of 0.63. While there are no aligned sign detections in the first iteration, the ratio of aligned detections increases fast and then levels off. Over the course of training, exploration is gradually replaced by exploitation: The more the sign detector improves, the more raw sign detections are available that are suitable for alignment.

### Effect of available annotations

**Fig 6:**
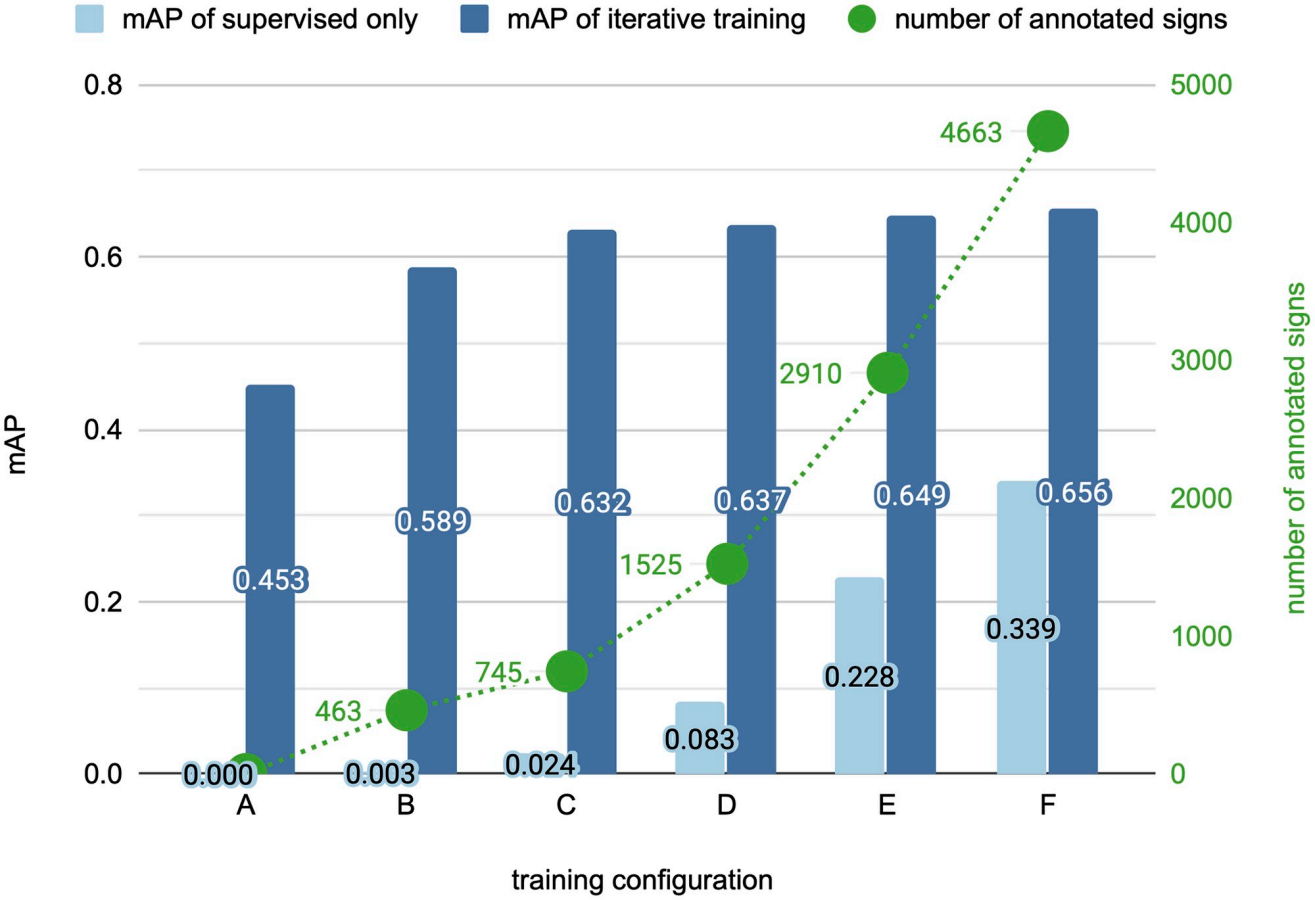
Effect of manual sign annotations on detection performance comparing purely supervised and our iterative training. There are six training configurations which differ in the number of available annotations. Configuration A corresponds to the weakly supervised case of iterative training. Configurations B to F correspond to the semi-supervised case. The detection performance (mAP) is reported on the test set.

In our third experiment, we evaluate the influence of manual sign annotations on the performance of the sign detector. The performance of a sign detector, after iterative training has converged, is compared with the performance of a sign detector after purely supervised training on manual sign annotations. We consider six different training configurations with zero (A), 463 (B), 745 (C), 1472 (D), 2910 (E) or 4663 (F) manual sign annotations respectively. For our iterative learning approach, configuration A corresponds to weakly supervised training and configuration B–F correspond to semi-supervised training. Similar to Fig 5, we train the weakly supervised case for two iterations and the semi-supervised cases for five iterations. The results are visualized in Fig 6. Overall the detection performance improves with additional manual annotations as expected. However, our iterative training significantly outperforms purely supervised training across all settings. Even if 4663 manual annotations are available (F), our iterative training surpasses supervised training by a large margin (65.6 mAP compared to 33.9 mAP). Using our iterative learning approach, the dependency on supervised training data is strongly reduced. Already the semi-supervised setting with 745 annotations (C) produces a sign detector performance that would require ten thousands of manual annotations in the purely supervised case.

### Qualitative detection results

**Fig 7:**
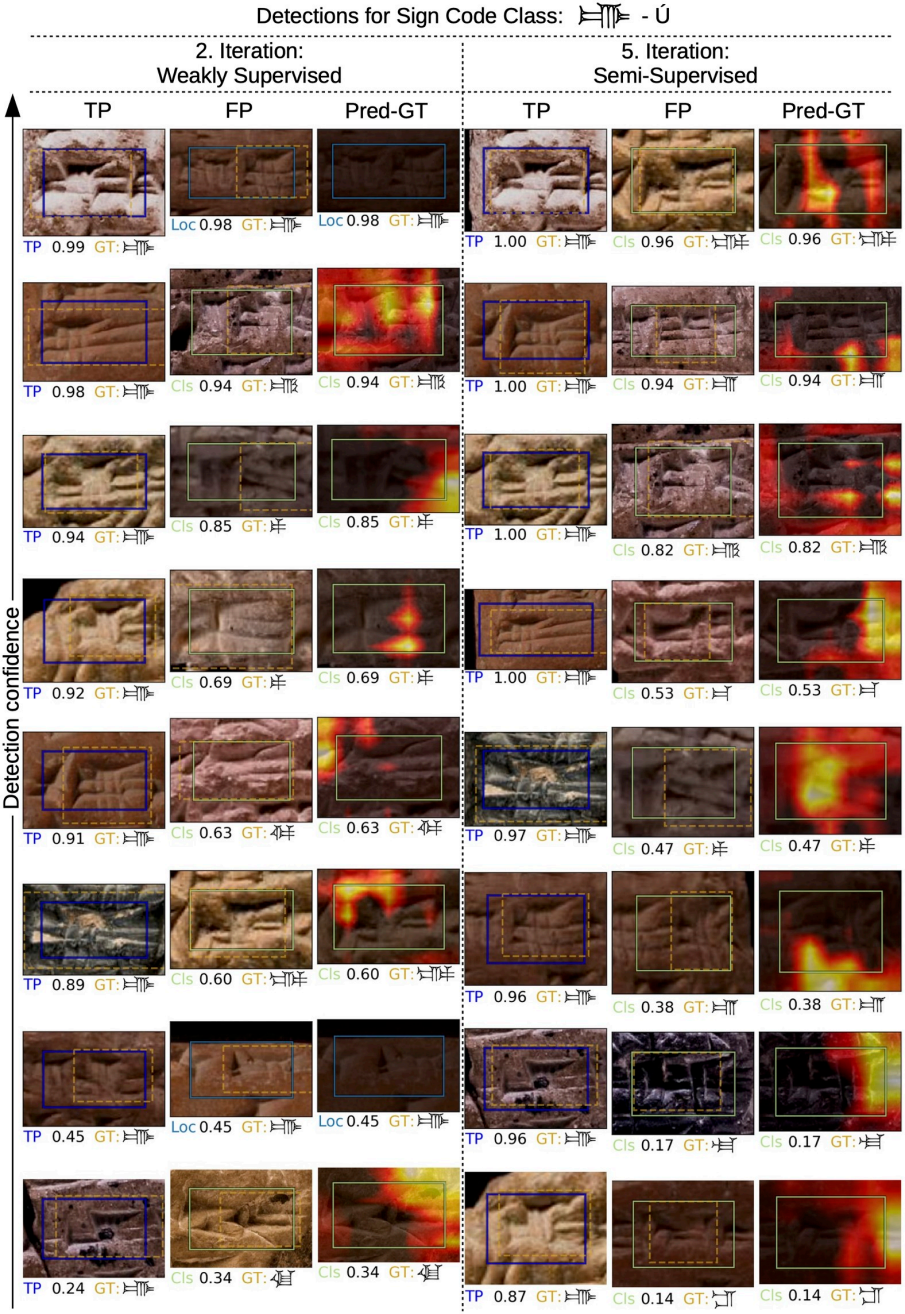
Individual sign detections for single sign code class at two different iterations of iterative training. For each iteration we show in the first two columns the eight most confident TP and FP sign detections on our test set. Each sign detection is represented as a solid bounding box, while the golden dashed box indicates the ground truth annotation (with the largest overlap with the predicted box). Below each detection we show the detection category, the detection confidence, and the actual ground truth (GT) sign code class. The detection category is either TP or one of three FP categories: Loc, BG, Cls. The third column of each iteration visualizes for each FP detection of the second column which image area contributed most to the detection error. Despite high intra-class variance and inter-class similarity of cuneiform signs the sign detector produces good results, makes plausible mistakes, and improves further in the semi-supervised case. Image material by the authors.

In our final experiment, we analyze individual sign detections and gain insight into the detector’s decision making. Fig 7 depicts detection results for a single sign code class (MZL no. 490) using a sign detector at two different iterations of iterative training. We apply the trained sign detector of the second experiment after the 2nd and 5th iteration to the test set. The resulting sign detections are classified as TP or FP based on ground truth annotations. The eight most confident (according to the sign detector) TP and FP detections are shown in the columns of Fig 7 sorted by their confidence value. Further, we follow the analysis as introduced in [45] and classify FP detections into three categories: Localization error (Loc), background confusion (BG) and similar class confusion (Cls). As expected the detection quality and confidence increases from 2nd to 5th iteration of iterative training which is visible in the TP columns. From studying the FPs, we find that the later detector makes more reasonable mistakes. In particular, sign localization is improved in the detector of the 5th iteration and all remaining FPs are due to class confusion, where predicted class and actual ground truth class look very similar.

Finally, we analyze which visual features are captured by the learned representation of the sign detector when it decides on the sign code class of cuneiform signs. We employ a technique called grad-CAM [47] that maps feature activations of the detector network back to the image domain. This back-projection can be conditioned on a specific sign code class in order to highlight most relevant image regions (support) for the sign code class in a heatmap. Since in the case of FPs the false predicted class and the actual ground truth class are known, we can go further in our analysis and focus on the difference between the support of the predicted class (Pred) and the support of the ground truth class (GT). The heatmap of this difference highlights the regions that exclusively contribute to the classification error according to the sign detector. In Fig 7 the third column of each iteration shows heatmaps with respect to the FPs from the 2nd column. In particular, the heatmaps of the 5th iteration of iterative training indicate where the detector hallucinates additional wedges in order to come to its FP prediction. Many mistakes appear plausible and are related to the difficulty of the fine-grained detection problem. There is no heatmap in the case of a localization error because the predicted and ground truth class are identical. See Figs G–K of S1 File for additional qualitative detection results including full tablet images.

## Discussion

In this article we have investigated how to train a cuneiform sign detector with minimal supervision. Instead of requiring manual annotations, our approach generates sign annotations for sign detector training by leveraging existing transliterations that are part of the Assyriologist’s workflow. In each iteration our iterative learning method alternates between training a sign detector and generating sign annotations by solving the alignment problem between tablet image and transliteration across hundreds of clay tablets in parallel. To improve the sign detector, our approach focuses on increasing the quality and quantity of generated sign annotations. To deal with the fine-grained nature of cuneiform sign detection, the strong imbalance of sign code class frequency and the difficulty of the transliteration alignment, we split the sign annotation generation into two distinct steps: sign placement and image-transliteration alignment. In this way we balance between learning from placed sign detections with sign code classes that are hard-to-predict or even unknown to the detector (exploration) and learning from aligned detections that are reliable (exploitation). The exploration part of our approach injects sign annotations with new sign code classes into the training of the sign detector by systematically placing signs from the transliteration in the gaps left after the alignment step. The exploitation part of our approach produces very reliable sign annotations that are consistent with the available bottom-up information (line and sign detections in tablet image) and top-down information (transliteration and line-geometry prior).

The performance analysis of the trained sign detector uncovers some of its limitations. The sign detector struggles with some rare sign code classes (see Sect. C1 of S1 File). Although the sign placement step explores new sign code classes and refreshes the memory of previously learned classes during iterative training, it cannot fully replace the collection of additional training data for rare sign code classes. Fortunately, only a few manual annotations are necessary to boost the sign detection performance thanks to weakly supervised learning, as shown in our third experiment. Additionally, to further reduce the burden of manual annotation, the purely weakly supervised sign detector can be leveraged to provide its sign detections to the annotator in a human-in-the-loop manner [48]. Instead of annotating tablet images from scratch, the annotation process is simplified to correcting the erroneous sign detections, i.e. changing labels and fixing bounding boxes. While the provided web application offers a basic implementation of this approach (see Sect. C8 of S1 File), it could be further refined using active learning strategies [49]. Even though the detector handles significant variations in writing styles, detection performance suffers from large variations in tablet (text) orientation, camera angle and image scale (image transformations). In this article we make the assumption that most available tablet images are properly orientated, which allows us to focus on sign detection performance independent of large image transformations. To increase the invariance to image transformations like rotation and scaling, we can use additional data augmentation during detector training and additional scale levels in the feature pyramid network of the sign detector. Of course, this additional invariance comes at a price of larger model size (more parameters) and increased training time.

We think that weakly supervised machine learning is an important research area that can profit from the study of such a challenging fine-grained detection problem. Research into object detection is often confronted with problems like object occlusion, object overlap, object viewpoint change and object class confusion. Cuneiform script provides a unique combination of these problems that are usually only available in separate object detection datasets: many tightly packed signs, fine-grained class differences, signs that are made up of 3D wedges that are hard to recognize due to viewpoint or damage, language-related dependencies between signs, and the geometric regularity of written script. By providing access to a new dataset with thousands of clay tablet images, transliterations and a bounding box annotated subset for evaluation, we hope to inspire researchers to explore the limits of fine-grained object detection and weakly supervised learning in the context of noise and ambiguity of cuneiform script.

While solving a challenging computer vision problem, our learning approach also provides a powerful tool for Assyriology. With a cuneiform sign detector, an important step of reading cuneiform script is significantly simplified. The resulting sign-by-sign representation together with bounding boxes provides a detailed analysis of cuneiform script in a clay tablet that is easy to interpret by a user. Our sign detector can be deployed in many situations, since it only requires 2D images as input that any smartphone camera is ready to produce.

However, in some cases detecting cuneiform signs correctly is impossible without additional context information. Assyriologists naturally take additional context knowledge about a clay tablet into account in order to arrive at their analysis. They look at the clay tablet from multiple viewpoints, remember similar texts, have a syntactic and semantic understanding, integrate knowledge from language, history and culture. The proposed cuneiform sign detector is only taking into account the direct neighbourhood of a cuneiform sign in the form of an image crop, when inferring the sign code class and its bounding box. While the context understanding of an Assyriologist is still beyond the-state-of-the-art of machine learning, we would like to take steps in this direction in future research. For example, a sign detector might fuse detections from 2D images from different angles of a clay tablet in order to deal with difficult cases. Similarly, if language models for cuneiform script become available in the future and problems like data sparsity are overcome [50], their integration will also help with ambiguous detections.

Our weakly supervised approach to learn a sign detector is not limited to cuneiform script of the Neo-Assyrian era. It is applicable to other cuneiform scripts like Old-Babylonian cuneiform as shown in Sect. C7 of S1 File. Our approach has been designed to deal with large discrepancies between tablet images and transliterations which is a common feature of ancient scripts and thus might provide a template for weakly supervised sign detection in other transliterated scripts.

Digital library projects like CDLI [17] and ORACC [31] grant researches online access to thousands of clay tablets and their transliterations, and offer new opportunities for large-scale digital analysis of cuneiform script. For this purpose, a sign detector provides a fast preliminary analysis of unstudied clay tablets. Additionally, the learned feature representation of a sign detector can serve as a similarity metric for the shape of cuneiform signs. Eventually, this will facilitate the visual search across thousands of tablet images and thus the reconstruction of broken tablets by speeding up the search for matching pieces.

## Supporting information

S1 File PDF file with supporting information. **A** Methods: A1–A9. Detailed description of the iterative training procedure including the sign detector training, placement and alignment steps, details on the network architecture for the line segmentation and sign detection networks, details on the training and evaluation of the presented experiments, and details on the software and hardware dependencies. **B** Sign code classes: B1–B2. **C** Sign detector performance: C1–C8. A performance analysis of the sign detector with quantitative results for individual sign code classes as well as qualitative results on full tablet images, details on the performance of different network architectures as well as pre-training, details about an experiment on Old-Babylonian cuneiform script, and details about the web application of the cuneiform sign detector.
(PDF)

S1 Video Demonstration of cuneiform sign detector in a web application. The purpose of this demonstration is to illustrate how a sign detector could be made available to Assyriologists. Through a web interface, the sign detector is applied to a tablet image. The detection results are immediately available for analysis by the user. Additional details regarding the demo application can be found in Sect. C8 of S1 File.
(MP4)
