# Supplementary material for: Deep learning of cuneiform sign detection with weak supervision using transliteration alignment
Source: PLoS One. 2020 Dec 16;15(12):e0243039. doi: 10.1371/journal.pone.0243039 (PMC7743970; doi:10.1371/journal.pone.0243039)
Supplement: S1 File — A Methods: A1–A9. Detailed description of the iterative training procedure including the sign detector training, placement and alignment steps, details on the network architecture for the line segmentation and sign detection networks, details on the training and evaluation of the presented experiments, and details on the software and hardware dependencies. B Sign code classes: B1–B2. C Sign detector performance: C1–C8. A performance analysis of the sign detector with quantitative results for individual sign code classes as well as qualitative results on full tablet images, details on the performance of different network architectures as well as pre-training, details about an experiment on Old-Babylonian cuneiform script, and details about the web application of the cuneiform sign detector. (PDF) [file pone.0243039.s001.pdf]

Deep learning of cuneiform sign detection with weak supervision using transliteration alignment

SUPPORTING INFORMATION

Tobias Dencker[1*] **Pablo Klinkisch**[1] **Stefan M. Maul**[2] **Björn Ommer**[1]
1 Heidelberg Collaboratory for Image Processing, Heidelberg University, Heidelberg, Germany
2 Institute for Assyriology, Heidelberg University, Heidelberg, Germany
tobias.dencker@iwr.uni-heidelberg.de

Supporting information A: Methods

In the following we provide additional details on all components of the iterative learning procedure that allows to train a cuneiform sign detector as summarized in Algorithm 1.

Algorithm 1: Weakly Supervised Iterative Training of Cuneiform Sign Detector

Input: L: Line detections; I: Tablet images; T: Transliterations; [B_{manual}: Manual sign annotations]
Output: D: Cuneiform sign detector
$B_{placed} \leftarrow \emptyset$; $B_{raw} \leftarrow \emptyset$; $B_{aligned} \leftarrow \emptyset$ `// Initialize placed, raw and aligned sign detections`
$A_{line} \leftarrow \emptyset$; $A_{sign} \leftarrow \emptyset$ `// Initialize line-level and sign-level alignment information`

Iterative Learning:
repeat
  1. SIGN PLACEMENT `// Sect. A3`
  if *first iteration* **then**
    $B_{placed} \leftarrow$ `LocalizeAllSigns`(T, L)
  else
    $B_{placed} \leftarrow$ `LocalizeUnalignedSigns`$(T, L, B_{aligned}, A_{line}, A_{sign})$

  2. SIGN DETECTOR TRAINING `// Sect. A4`
  $D \leftarrow$ `TrainNewSignDetector`$(I, B_{aligned} \cup B_{placed}, L)$
  if B_{manual} *is available* **then**
    $D \leftarrow$ `FineTuneSignDetector`(D, I, B_{manual}) `// semi-supervised case`
  $B_{raw} \leftarrow$ `RunSignDetection`(D, I)

  3. IMAGE-TRANSLITERATION ALIGNMENT `// Sect. A5 (line-level); Sect. A6 (sign-level)`
  $A_{line} \leftarrow$ `OptimizeLineLevelAlignment` (T, L, B_{raw})
  $[B_{aligned}, A_{sign}] \leftarrow$ `OptimizeSignLevelAlignment`$(T, L, A_{line}, B_{raw})$

until D *is converged*

A1 Iterative Training with Weak Supervision

Before the start of the iterative training, the line segmentation network is trained on a small set of labeled tablet images and applied to all tablet images of the train set in order to obtain line detections (Sect. A2). The iterative training generates in each iteration a new set of aligned and placed detections that is used to supervise the training of the cuneiform sign detector (Sect. A4). The iterative training, summarized in algorithm 1, proceeds as follows: 1) Sign placement produces placed detections using transliteration and line detections in the first iteration and later also incorporating aligned detections

[*]To whom correspondence should be addressed.

(Sect. A3). 2) We train a new sign detector on the union of aligned and placed detections, and fine-tune it on manual sign annotations if available (Sect. A4). 3) We apply the trained sign detector to the Train TL set (unlabeled tablet images) and use the line and sign detections to find an image-transliteration alignment on line-level (Sect. A5). Then for each line we find a sign-level alignment between sign detections and transliteration vector in order to produce a set of aligned detections (Sect. A6). The iterative training of steps 1-3 is repeated until the performance of the sign detector stops improving. The fine-tuning step is the only difference between the weakly and semi-supervised case of our learning approach.

A2 Line Detection

Our line detection consists of two steps: 1) Line segmentation with a convolutional neural network (CNN) and 2) Hough transform-based post-processing to obtain line detections. For line segmentation we train a CNN to classify image patches as line center or background. During training we sample image patches close the boundary of line annotations more frequently, thus focusing the classifier training on hard-positive and hard-negative cases. At test time, we obtain a segmentation map of the full image by turning the classifier into a fully-convolutional network. As CNN architecture we use a modified AlexNet [1] with BatchNorm after convolutional layer 3-5 and only one linear layer with 512 neurons before the classification head.

As training data for the line segmentation network (line center classifier), we manually annotated all visible lines of cuneiform script in 38 segmented views of clay tablets. In total we obtain 410 annotated lines of cuneiform script for training. A line is annotated as a sequence of connected linear segments (piece-wise linear segments) by marking points on the line: the start, the end, and points in between to cover curved text. Most line annotations consist of four linear segments (five points).

For robust line detection, we post-process the line segmentation using the straight line Hough transform [2]. By identifying the peaks in the Hough transform, we obtain votes for the presence of lines in the tablet image. We expect lines on the same tablet to be similar oriented and have a minimum distance to each other, therefore we require detected lines to be close in orientation (+/- 3 degree), but far enough apart vertically (minimum 50 pixels). We use the median angle of all lines (peaks in Hough transform) that we find in a first run in order to re-focus the line search on a tighter range of line orientations in a second run and thus avoid outliers. Further, we merge lines if they intersect or are almost parallel. Finally, detected lines are associated with their segmentation mask which can provide additional information.

A3 Sign Placement Method

In the first iteration of iterative learning, the sign placement hypothesis (placed detections) is purely created from line detections and sign size statistics, and thus, serves as initial training data for the cuneiform sign detector. In later iterations, the sign placement method additionally leverages aligned detections in order to localize unaligned transliterated signs with higher precision.

To generate the initial placed detections, we first align transliteration lines with detected lines by matching them in a greedy fashion from top to bottom, i.e. the first translation line is assigned to the first detected line etc. If aligned detections are available, we rely on the assignments of the line-level alignment method as described in Sect. A5.

Having solved the line-level alignment, the placed detections are generated line by line. For the sign placement method, a line is defined as the linear segment between a start and end point, which are localized in the tablet image with help of the line detections and its segmentation mask. If aligned detections are available, they provide additional reference points for sign placement besides the start and end of a detected line. Aligned detections effectively split a full line into smaller individual line segments of unaligned transliterated signs whose start and end points are the respective borders of the bounding box of the aligned detections. Knowing start and endpoint of a line segment, we place the center points of corresponding transliterated signs on the line so that they span the full length of the line. The bounding box size of each placed detection is estimated in the following way: The sign height is estimated from the average line distance in the tablet image. Since sign widths strongly vary across sign code classes, we compute the sign width by multiplying a class-specific normalized sign width from a pre-computed sign size statistic with the sign height. We collect the sign size statistic by measuring the relative length and width of sign characters in a realistic cuneiform Unicode font [3].

If aligned detections are available, we additionally filter the resulting placed detections twofold in order to increase their precision: 1) Placed detections that are more than three signs distant from the nearest line start or end point (e.g. aligned detection) are ignored. 2) Placed detections are only included, if at least two aligned signs are present in their line.

A4 Sign Detector Training

We implement the SSD detector [4] with default boxes that cover four aspect ratios (3/5, 1/1, 2/1, 3/1) and three scales (1, 1.26, 1.59) that are adjusted for the various shapes of cuneiform signs. During training we use online hard-negative mining as described in [4] which maintains an one-to-three ratio of positive and negative boxes by keeping only the hardest negatives.

Similarly, we follow in their choice of loss functions by using a cross-entropy loss for the classification head and a smoothed L1 loss for the bounding box regression head. In the implementation of the feature pyramid network, we deviate from [5] and only detect cuneiform signs across two feature scale levels that assume signs to fit in a window (anchor box) of 128×128 or 256×256 pixels, respectively. We do not search for cuneiform signs across all feature levels, since after pre-processing tablet images are resized to match a sign height of 128 pixels as described in Sect. A7.

When training the sign detector, we always pre-train the backbone network on the simpler task of sign code classification and then use the pre-trained backbone as initialization for detector training. For this pre-training the backbone network is extended with an average pooling layer, followed by a liner layer with as many neurons as classes and a softmax function that provides class predictions. The training of the sign code classifier on the generated sign annotations from our weakly supervised approach is performed like training on supervised data, in contrast to the weakly supervised training of the sign detector.

Standard training of an object detector requires fully annotated images, however, the placed and aligned detections only cover a subset of all visible signs in tablet images. We use the line segmentation mask to prevent the incorrect labeling of foreground bounding boxes as background (false negatives) and to include many true hard negatives found at the border of the mask for training. In particular, bounding boxes are ignored, if both of the following conditions hold true: 1) The box center is located on a segmentation mask of a detected line, and 2) the maximum intersection-over-union (IoU) with any aligned detection box is in the range $[0, 0.35)$. For all other boxes the standard rules for detector training apply.

After training on weakly-supervised data, the sign detector can be fine-tuned on manual sign annotations which represents semi-supervised training. Annotated bounding boxes provide accurate sign localization also in difficult cases and thus mitigate the problem of localization drift as shown in Fig. F. If all signs in an image are annotated, no masking based on the line segmentation is required which further increases the quality of hard negatives available for training.

A5 Line-level Alignment

Given the detected lines in the tablet image and the lines in the transliteration, the goal is to find the correct line-level alignment. This optimization is necessary because of errors in line detection (e.g. false positives) and the discrepancy between tablet image and transliteration (e.g. transliteration contains signs that are not visible in image and vice versa).

We draw inspiration from the sentence alignment problem in natural language processing, and thus adapt the Bleualign algorithm [6] which formulates the alignment problem (for sentences in text a and its translation b) as a longest path search. For all combinations of detected lines and transliteration vectors (transliterated lines), an alignment score is computed and stored in a score matrix. The rows and columns of the score matrix correspond to detected lines and transliterated lines sorted by line number in descending order. We construct a directed grid graph G in the size of the score matrix, where diagonal edges resemble “matches” and horizontal or vertical edges “skips”. The alignment is obtained by optimizing the path in G from the upper left to the lower right node. The alignment scores from the score matrix define the cost of matches and skips implied by the selected path. Since G is a direct acyclic graph (DAG) with positive edge weights the optimization is performed in linear time using the topological sort algorithm.

The original Bleualign algorithm makes use of a machine translation system to map a source sentence into the language of the target sentence, before computing their BLEU [7] score as alignment score. In the case of line-level alignment we need to associate a detected line in image-space with a transliteration vector in sign-code-space. For each detected line we identify a line-specific subset of the raw detections by selecting only detections close to the detected line (less than half the average line distance). When we sort the remaining sign detections according to their horizontal position to obtain the predicted transliterated line in sign-code-space for which we can compute a matching score.

Besides the BLEU score, we consider alternative alignment scores that are not only dependent on the output of our sign detector, but also incorporate geometric constraints. In particular we derive a score from the result of the sign-level alignment method which we describe in following Sect. A6. Having computed paths from two different score matrices, we take the intersection of both paths in order to retain the most robust line-level alignment.

A6 Sign-level Alignment

For a given the line-level alignment between a detected line and its corresponding transliteration, the goal of sign-level alignment is to find an assignment between the raw detections in the tablet image and the signs in the transliteration vector. We follow the idea of part-based graphical models [8] and build a conditional random field (CRF) model for a line, whose underlying graph structure is determined by the transliteration vector.

Formally, we represent the transliteration vector T with n signs as a fully connected graph $G = (V, E)$ as shown in the example in Fig. A. Each node $a \in V$ we associate with a sign $t_a \in T$ in the transliteration and a random variable X_a that

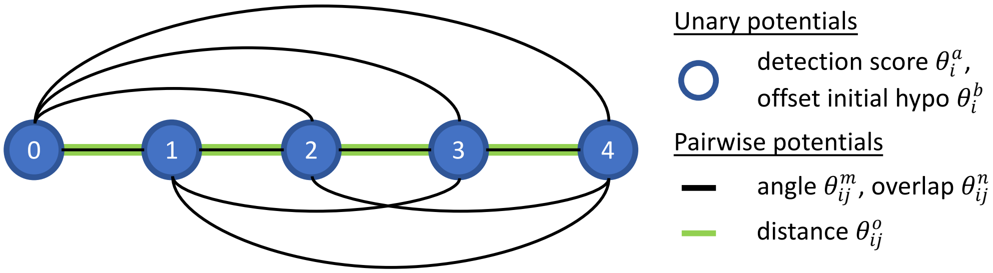

Figure A: The conditional random field (CRF) has a fully-connected graph which models appearance and geometric constraints of a line. Each random variable (circled number) represents a sign in the transliteration (this figure shows the graphical model for a transliteration of five signs). There are unary potentials for each variable depending on detection score and offset from a initial sign hypothesis. Connections between variables describe pairwise potentials that enforce geometric constraints found in a line like angle, overlap and distance between signs. The distance potential (green connections) only affects connections of neighbouring signs.

takes values $x_a \in C_a$, where C_a is the set of detections of sign code class t_a. Additionally C_a includes a candidate ϵ that indicates a sign that could not be aligned. The alignment of a transliteration with sign detections is then given by the vector $\mathbf{x} = (x_a)_{a \in V} \in C^n$, where we use C^n to describe the set of all possible alignments (ie. assignments to all random variables). The energy function $\mathbb{E} : C^n \to \mathbb{R}$ of the CRF maps any alignment to a real number. The function $\mathbb{E}(\cdot)$ is defined as the sum of all unary $\theta^U(\cdot)$ and pairwise potential terms $\theta^P(\cdot,\cdot)$ as follows:

$$\mathbb{E}(\mathbf{x}) = \sum_{i \in V} \theta_i^U(x_i) + \sum_{(i,j) \in E} \theta_{ij}^P(x_i, x_j) \tag{1}$$

The unary terms θ^a, θ^b take into account the detection confidence and the offset from an initial sign hypothesis respectively, and are combined using weights λ_a and λ_b as follows:

$$\theta_i^U = \lambda_a \theta_i^a + \lambda_b \theta_i^b \tag{2}$$

The pairwise terms $\theta^m, \theta^n, \theta^o$ constrain the overlap between sign detections, distance between bounding boxes as well as angle between signs and detected line respectively, and are combined using weights λ_m, λ_n and λ_o as follows:

$$\theta_{ij}^P = \lambda_m \theta_{ij}^m + \lambda_n \theta_{ij}^n + \lambda_o \theta_{ij}^o \tag{3}$$

Unary terms For detector confidence we define the unary term $\theta_i^a(x_i) = \exp((1 - score(x_i))/\sigma_a) - 1$, where $score(\cdot)$ returns confidence of the assigned detection. For the offset from the initial sign placement hypothesis we define the unary term $\theta_i^b(x_i) = hypodist(x_i))$, where $hypodist(\cdot)$ returns the euclidean distance between the estimate position of the sign from the initial hypothesis and the location of the assigned detection. We initial sign hypothesis is generated by the sign placement method described above.

Pairwise terms For the overlap between sign detections we define the pairwise term $\theta_{ij}^m(x_i, x_j) = \exp(iou(x_i, x_j)/\sigma_m) - 1$, where $iou(\cdot,\cdot)$ computes the intersection-over-union between the bounding boxes of the assigned detections. For the distance between bounding boxes we define the pairwise term $\theta_{ij}^n(x_i, x_j) = \exp(boxdist(x_i, x_j)/\sigma_n) - 1$, where $boxdist(\cdot,\cdot)$ computes the distance between the bounding boxes of the assigned detections. The potential is only nonzero for connections between neighbouring signs (green connections in Fig. A). For the angle between signs and detected line we define the pairwise term $\theta_{ij}^o(x_i, x_j) = \exp(angle(x_i, x_j)/\sigma_o) - 1$, where $angle(\cdot,\cdot)$ computes the angle of the vector that connects the bounding boxes of the assigned detections and the vector of the detected line.

Outlier treatment If a random variable X_a takes on the value $x_a = \epsilon$, it incurs a fixed outlier penalty λ_p independent of any neighbouring nodes. This outlier class deals with difficult alignments (e.g. no matching detection).

Inference After computing the unary and pairwise potentials for a given line, we use the sequential tree-reweighted message passing (TRW-S) algorithm [9] to minimize the energy function in (1). The solution aligns a detections with each sign in the transliteration vector except for signs that have been assigned to the outlier class. Our implementation makes use of the OpenGM framework [10].

A7 Training and Evaluation

All neural networks of our approach are trained using a standard stochastic gradient descent (SGD) optimizer with momentum 0.9 and weight decay of 1e-04. The configuration of learning rates (lr) and other parameters is described in the following.

Pre-processing of Tablet Images First, we convert all tablet images to gray-scale and compute line detections using our method. Then we resize the tablet images so that the height of a cuneiform sign is about 128 pixels. We obtain the necessary scaling factor for each tablet image by estimating the average sign height. We approximate the average sign height with the average distance between detected lines. If there is a transliteration available, we also estimate upper and lower bounds for the sign height in the table image by dividing the tablet image height by the number of transliterated lines and by dividing the tablet image width by the length of the longest transliterated line respectively. If the estimated sign height is out of bounds, we use the nearest bound instead. To refine the line detections and sign height estimates, we re-run the steps of pre-processing once on the scaled version of the tablet images.

Sign Code Classification The sign code classifier is trained with lr 0.01, until the train error plateaus and then lr is decreased by factor 0.1. This is repeated two times. As input data 224x224 patches are randomly cropped from a sign bounding box that has been context-padded to 256x256 box without altering the aspect ratio.

Line Segmentation The line segmentation network is trained with lr 0.01 which is decreased like in the case of sign code classification training. As input data 227x227 patches are randomly cropped from 256x256 patch that is sampled following the strategy describe in Sect. A2.

Sign Detection The sign detector is always trained for 50 epochs with lr 0.001 with online hard-negative mining as described in [4]. As input data 512x512 patches are randomly cropped from a tablet image which has been split in 600x600 patches that overlap by 200px. As data augmentation we use randomly resized crops with scale range $[0.65, 1]$.

Detector Fine-tuning In the case of semi-supervised training, the regular training on weakly supervised data is followed up by a fine-tuning step on annotated samples. The sign detector is fine-tuned on the annotated samples for 20 epochs with a reduced lr 1e-04.

Alignment Method The configuration of λ and σ parameters defined in Sect. A6 is shown in Table A. The values in the brackets correspond to the parameter configuration of pairwise potentials between two non-neighbouring signs. Having aligned detections we compute for each tablet its alignment ratio (AR), the ratio between aligned detections and signs in transliteration. We only keep aligned detections from tablets with $AR > 0.3$ in order to focus on the most reliable alignments.

Evaluation In the case of sign detection, we follow the standard evaluation protocol for object detection. We use class-wise non-maximum suppression (NMS) with threshold at 0.3 intersection-over-union (IoU). A detection is considered true positive (TP), if the IoU between its bounding box and a ground truth box is larger than 0.5, otherwise it is a false positive (FP). Then we compute the mean average precision (mAP) across all 186 sign code classes.

Table A: The configuration of λ and σ parameters for the sign-level alignment in Sect. A6 used for all experiments.

outlier	score		hypodist	iou		boxdist		angle	
λ_p	λ_a	σ_a	λ_b	λ_m	σ_m	λ_n	σ_n	λ_o	σ_o
25	12	0.88	1	0.4 (1.5)	0.4 (0.05)	5	3	2 (0.2)	0.6 (0.1)

A8 Visualizing Detection Support

For the visualization of the sign support, we make use of the gradCAM method [11]. We visualize the feature activation in the penultimate layer of our adapted MobileNet-v2 network (see Sect. A4). We use absolute values for the resulting heatmap and normalize it in the range of $[0, 1]$. We first compute the support with respect to the predicted class $pred$ and ground truth class gt separately. Then we compute the difference of support between $pred$ and gt with the following function: $min(max(pred - gt, 0), 1)$.

A9 Software and Hardware Dependencies

Software We implemented our approach using the PyTorch deep learning library [12]. Details regarding our implementation including Python dependencies for installation are listed on the GitHub project page.

Hardware Training and evaluation are performed on a machine with a single GPU (we used a Gefore GTX 1080, a consumer GPU). The web application demo runs on a web server without GPU support, since detection inference with a lightweight MobileNetV2 backbone is fast even in CPU-only mode (less than 1s for an image with HD resolution, less than 10s with 4K resolution).

Supporting information B: Sign Code Classes

We provide additional details on the different cuneiform sign code classes in our dataset.

B1 Sign Code Classes used for Detection

The cuneiform sign detector is evaluated on 186 different cuneiform sign code classes that are present in the train set and have at least one annotated example in the test set. Fig. B illustrates the fine-grained nature of this detection task. The diversity as well as the similarity between sign code classes is apparent by just comparing the characters printed in a Unicode cuneiform font [3]. We show the cuneiform signs sorted according to their sign code as introduced in Borger's sign listing [13], which groups signs according to common substructures and similarity. Often cuneiform signs are composites of several smaller signs, where the only difference between two classes is a single wedge. Besides high inter-class similarity the variation between signs of the same class in real tablet images is high as well as we will illustrate in Fig. G.

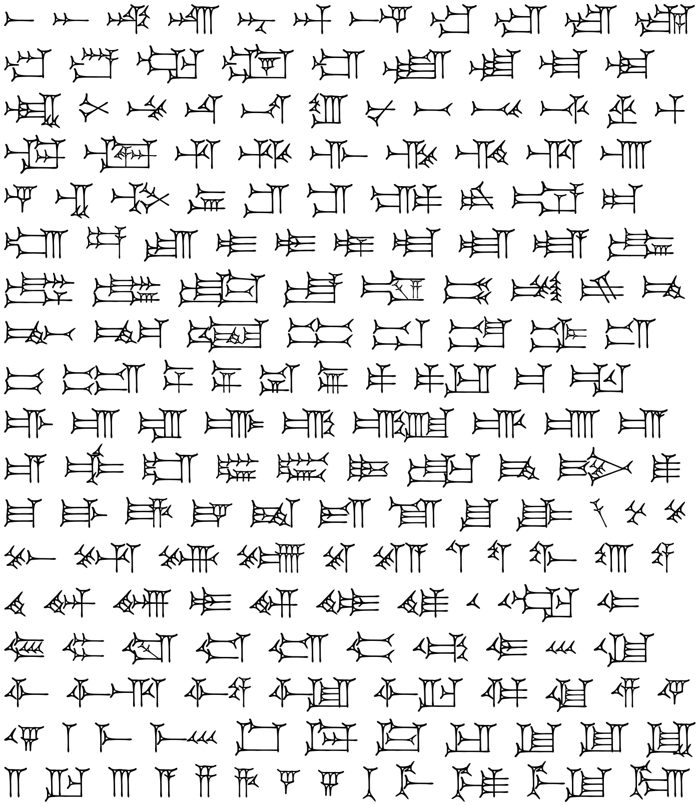

Figure B: Unicode characters of sign code classes considered for detector training. We show all cuneiform signs which are supported by the Unicode font (181 out of 186).

B2 Frequency of Sign Code Classes

Fig. C visualizes the frequency of the 186 sign code classes in Train TL set (unlabeled tablet images with transliteration). We only count an occurrence of a sign in Train TL, if it is not labeled as broken in the transliterations. The resulting frequency distribution roughly follows a discrete Pareto distribution. When visualized in log-scale the exponential distribution becomes a linear trend. Further we can observe that the least frequent 25 sign code classes have less than 100 occurrences, whereas the most frequent 25 have more than 2000.

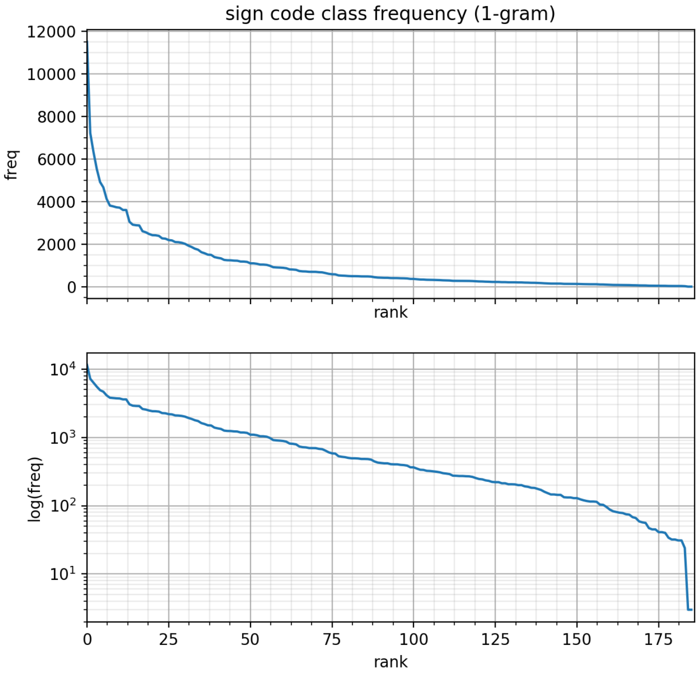

Figure C: Plot of the (1-gram) frequency of the 186 sign code classes in the Train TL set with classes ranked according to their frequency. In the upper plot the exponential drop in class frequency is visible, which indicates a discrete Pareto distribution. This is confirmed by the lower plot with logarithmic scale that shows a linear trend for most of the classes.

Supporting information C: Details on Detector Performance

We present results on the class-wise detection performance of our iterative learning approach, analyze the false positive errors of the sign detector, and show qualitative results on full tablet images as well as for individual sign detections. Moreover, we compare the impact of different backbone architectures as well as ImageNet pre-training, we explore if the learned representation improves sign detection of a different cuneiform script, and finally we showcase a web application of the cuneiform sign detector.

C1 Class-wise Detection Results

Fig. D reports the sign detector performance on individual sign code classes evaluated on the Test set in terms of class-wise average precision (AP). In contrast, the mean class AP (mAP) provides a metric for the average detector performance across classes. The results are based on the sign detector that we obtain after iterative training in a semi-supervised fashion using all manual sign annotations of Train BB for fine-tuning. The sign code classes are sorted according to their AP (in descending order). We show every second sign code class in the Test set to provide a broad overview of the class-wise sign detector performance, while not overfilling the page.

Since the different cuneiform signs have a strongly imbalanced class distribution (Pareto-distributed), we provide the frequency of signs in the Train TL, the Train BB and the Test set. One might expect that well performing classes usually belong to the group of signs that occur often in the dataset (high support). In Fig. E we analyze the correlation across the 186 sign classes between detector performance and the support in Train BB and the support in Train TL. We find a light positive correlation for the two properties, where sign code class frequency in Train TL seems to be more important than frequency in Train BB. While the sign detector seems to slightly favor more frequent signs, it still performs well for many less frequent signs.

#	MZL	Sign	TrainTL	TrainBB	Test	AP
0	899	[illegible]	41	1	1	**100.0**
2	756	[illegible]	79	4	3	**100.0**
4	246	[illegible]	41	4	3	**100.0**
6	698	[illegible]	213	6	6	**100.0**
8	540	[illegible]	56	4	2	**100.0**
10	223	[illegible]	386	21	3	**100.0**
12	544	[illegible]	96	4	1	**100.0**
14	65	[illegible]	47	1	1	**100.0**
16	362	[illegible]	206	6	1	**100.0**
18	585	[illegible]	81	2	1	**100.0**
20	127	[illegible]	696	25	2	**100.0**
22	726	[illegible]	153	5	4	**100.0**
24	543	[illegible]	129	7	2	**100.0**
26	238	[illegible]	206	8	5	**100.0**
28	143	[illegible]	192	6	2	**100.0**
30	747	[illegible]	295	9	5	**96.7**
32	856	[illegible]	672	20	20	**95.0**
34	635	[illegible]	417	14	11	**93.2**
36	16	[illegible]	698	22	16	**91.4**
38	136	[illegible]	866	15	11	**89.3**
40	560	[illegible]	886	19	11	**89.3**
42	350	[illegible]	1181	26	15	**88.6**
44	266	[illegible]	4913	39	16	**86.3**
46	869	[illegible]	2874	82	59	**85.0**
48	589	[illegible]	1096	43	33	**84.3**
50	339	[illegible]	299	3	10	**83.6**
52	252	[illegible]	3727	83	91	**83.3**
54	326	[illegible]	24	4	2	**83.3**
56	496	[illegible]	914	19	17	**81.3**
58	887	[illegible]	114	5	6	**80.8**
60	10	[illegible]	6310	190	116	**80.7**
62	142	[illegible]	1503	49	33	**79.8**
64	358	[illegible]	2012	50	29	**77.1**
66	681	[illegible]	584	5	8	**76.4**
68	552	[illegible]	4124	94	70	**76.1**
70	498	[illegible]	1865	43	42	**75.8**
72	110	[illegible]	5523	111	68	**75.5**
74	118	[illegible]	1785	35	32	**75.1**
76	745	[illegible]	223	6	9	**75.0**
78	754	[illegible]	3601	111	79	**74.9**
80	86	[illegible]	981	24	19	**74.2**
82	99	[illegible]	417	10	7	**73.3**
84	596	[illegible]	3601	60	65	**72.8**
86	14	[illegible]	1038	43	36	**72.6**
88	724	[illegible]	2061	40	100	**71.6**
90	558	[illegible]	78	3	6	**71.1**
92	380	[illegible]	7211	91	70	**70.1**
94	566	[illegible]	3708	65	46	**69.7**
96	24	[illegible]	2088	96	50	**69.1**
98	828	[illegible]	1024	14	16	**68.3**
100	599	[illegible]	1356	29	47	**67.5**
102	491	[illegible]	403	15	20	**67.2**
104	690	[illegible]	144	6	3	**66.7**
106	729	[illegible]	144	2	3	**66.7**
108	760	[illegible]	32	0	3	**66.7**
110	464	[illegible]	1735	42	45	**64.9**
112	884	[illegible]	290	2	11	**63.8**
114	469	[illegible]	1238	26	16	**63.7**
116	297	[illegible]	308	6	4	**62.5**
118	119	[illegible]	246	1	8	**61.5**
120	262	[illegible]	124	4	2	**61.1**
122	636	[illegible]	396	11	5	**60.0**
124	859	[illegible]	2254	62	46	**57.1**
126	180	[illegible]	200	6	7	**56.7**
128	258	[illegible]	805	19	14	**56.1**
130	541	[illegible]	182	8	10	**55.8**
132	5	[illegible]	231	7	3	**54.4**
134	591	[illegible]	272	5	2	**54.2**
136	15	[illegible]	200	18	6	**53.0**
138	644	[illegible]	334	11	7	**51.4**
140	353	[illegible]	254	4	19	**50.5**
142	6	[illegible]	132	3	2	**50.0**
144	9	[illegible]	274	9	13	**49.5**
146	113	[illegible]	2474	36	17	**48.6**
148	18	[illegible]	903	11	7	**48.3**
150	851	[illegible]	791	26	25	**46.1**
152	1	[illegible]	4663	129	87	**45.2**
154	261	[illegible]	349	5	6	**44.5**
156	92	[illegible]	530	17	37	**43.6**
158	474	[illegible]	405	5	7	**42.9**
160	579	[illegible]	604	13	12	**42.2**
162	302	[illegible]	146	3	16	**38.6**
164	631	[illegible]	1179	26	21	**37.3**
166	746	[illegible]	88	3	3	**35.7**
168	755	[illegible]	104	3	3	**31.1**
170	686	[illegible]	40	2	2	**25.0**
172	222	[illegible]	120	1	1	**20.0**
174	167	[illegible]	115	2	4	**18.8**
176	720	[illegible]	235	4	1	**7.1**
178	825	[illegible]	697	13	4	**3.4**
180	640	[illegible]	264	5	4	**2.3**
182	632	[illegible]	59	6	1	**0.0**
184	733	[illegible]	75	9	1	**0.0**

Figure D: Class-wise detection results showing every second sign code class sorted according to sign detector average precision (AP) in descending order. For each sign code class we report the following: corresponding sign code (MZL) as used in Borger's sign lists [13], Unicode character (Sign), frequency of occurrence in Train TL, Train BB (with bounding boxes) and Test set, as well as the corresponding AP of the sign detector on the Test set.

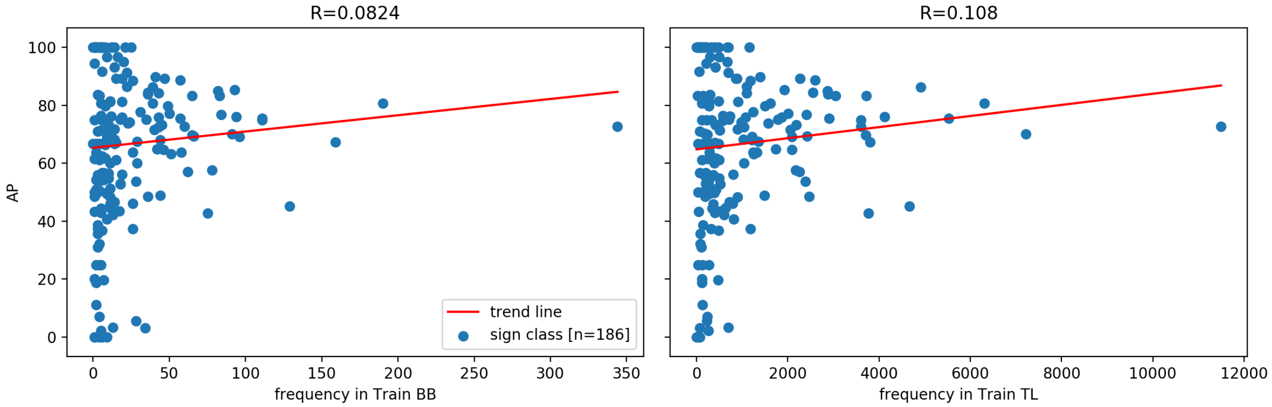

Figure E: Two scatter plots studying potential correlation between performance of sign detector on individual sign code classes and their frequency in the Train TL and their frequency in Train BB (from left to right respectively). The red curve is based on a linear regression indicating the data trend and the number on top of each plot is the Pearson correlation coefficient.

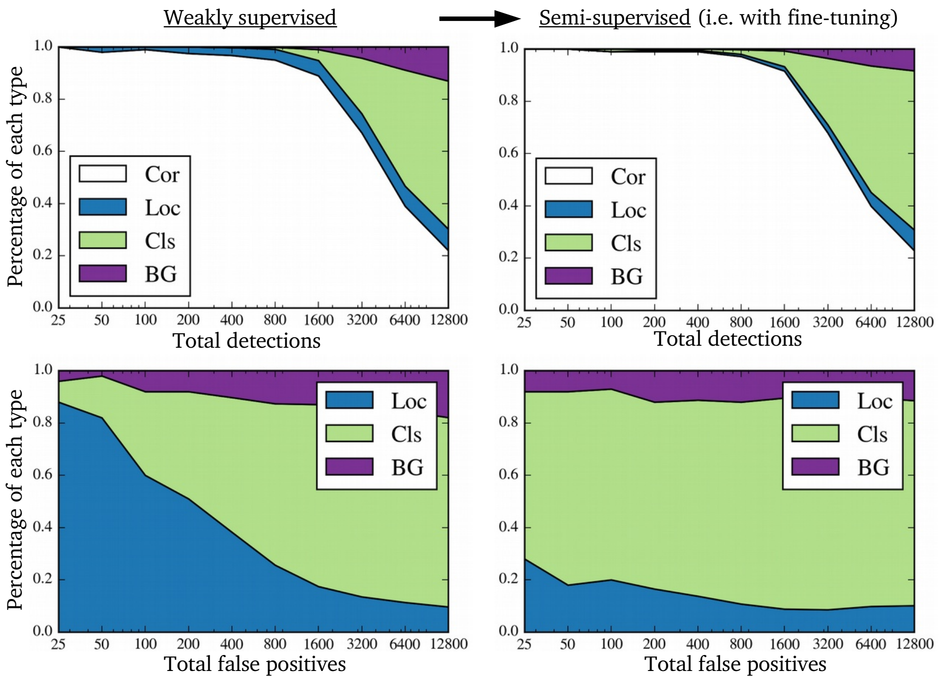

Figure F: False positive (FP) error analysis of sign detector on test set. In each plot the detections are ranked according to their confidence along the x-axis (starting with highest confidence from the left). Along the y-axis the distribution of detection types (Cor: true positive, Loc: localization error, Cls: class confusion, BG: background confusion) is visualized. Top row: All detections including true positives (Cor). Bottom row: Only FP detections. Left column: Weakly supervised training causes a high percentage of localization errors w.r.t. all FPs. Right column: Fine-tuning mitigates the impact of badly localized detections significantly.

C2 False Positive Analysis

False positive (FP) categorization provides helpful insights into the performance of an object detector. We follow the analysis as introduced in [14] in order to classify false positives into three categories: Localization error (Loc), background confusion (BG) and similar class confusion (Cls). Fig. F shows the false positive analysis for a sign detector on the test set evaluated at two different stages of sign detector training.

When training the sign detector with weakly supervised data, badly localized bounding boxes can cause an increase of localization error as shown in the false positive category distribution in the left column of Fig. F. The right column shows the false positive category distribution of the same sign detector after fine-tuning on manual annotations. Fine-tuning results in a significant decrease of localization error which is visible in the difference between the left to right column. The fine-tuned detector produces a large number of true positives (TP) detections and reduces localization and background errors. The similar class confusions make up the majority of detection errors due to the fine-grained differences between cuneiform signs.

C3 Individual Sign Detections

In Fig. G we plot individual true positive sign detections for two very similar-looking cuneiform sign code classes. Besides the high inter-class similarity between the different columns in Fig. G, there is also a high intra-class variance visible across the different sign detections of each column. Even signs of the same class and from the same tablet (same scribe) can vary in appearance considerably. Multiple factors contribute to intra-class variance and are visible across the sign detections of each column: Changing writing styles, overlapping signs, differences in the the clay material, illumination, orientation of the tablet to the camera, and the state of conservation of each sign. The sign detector trained by our weakly supervised approach is able to deal with this challenging setting and demonstrates good results on a diverse set of tablet images.

C4 Detection Results on Tablet Images

Fig. H and Fig. I visualize qualitative detection results on full tablet images that are not part of the train set after iterative training (weakly supervised and semi-supervised with 745 manual sign annotations). We color-code TP and FP detections using the ground truth sign annotations (bounding boxes). In Fig. H relatively few errors are visible. Errors tend to occur

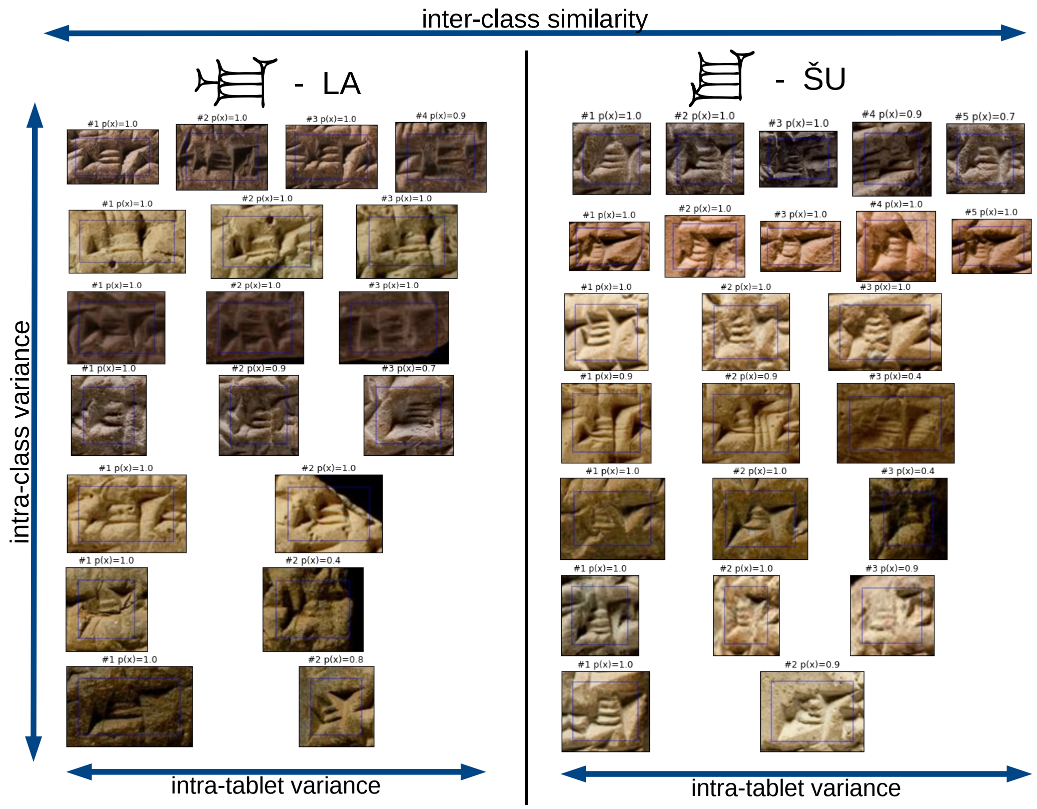

Figure G: True positive detections of two different cuneiform sign code classes arranged in two columns. For each column each row of detections is obtained from a single tablet image. The blue box depicts the detected bounding box. Above each detection its confidence is reported.

more often on the border of the tablet which is mostly due to curvature of the tablet as well as damaged or broken signs. The examples in Fig. I illustrate levels of damage that are common in the SAAo dataset. Despite of this, the sign detector provides robust detections and many errors correlate with badly damaged signs.

Fig. J and Fig. K visualize qualitative detection results on full tablet images from the test set over three iterations of weakly supervised and two iterations of semi-supervised training (see main article for quantitative results of this experiment). The performance improvement over the course of iterative training is clearly visible. The most prominent changes occur in earlier iterations, while later changes are more subtle (e.g. improvements in localization and rare classes).

C5 Backbone architecture comparison

A deep neural network-based sign detector is composed of a backbone and a detection head network. The backbone network is the core of the learned representation which is usually pre-trained on a classification task. In Fig. L we compare four different backbone architectures on the task of sign code classification (with 186 classes) on our test set. The classification performance of a backbone architecture provides a decent indicator for the overall detection performance [15]. All configuration have been trained on the train set E using a SGD optimizer with momentum. Learning rate and training schedule have been manually tuned for best performance. The network performance is reported as percentage of correctly classified signs (classification accuracy). The number of learnable parameters of the backbone networks is also visualized, since this is an important factor for inference speed, hardware requirements and model regularization. We compare two MobileNet-v2 [16] based architectures and the well-known AlexNet [1] and ResNet-18 [17] architectures. The *cuneiform* version of MobileNet-v2 is our adapted version (see Sect. A4) used in all our experiments whereas the *original* version implements the exact architecture of [16] with its width multiplier set to 0.75. The *cuneiform* version of MobileNet-v2 is by far the smallest backbone architecture with about 700K parameters as plotted in Fig. L. Nevertheless it shows competitive performance when compared to the other models. Using the larger *original* version of MobileNet-v2 improves performance. The AlexNet

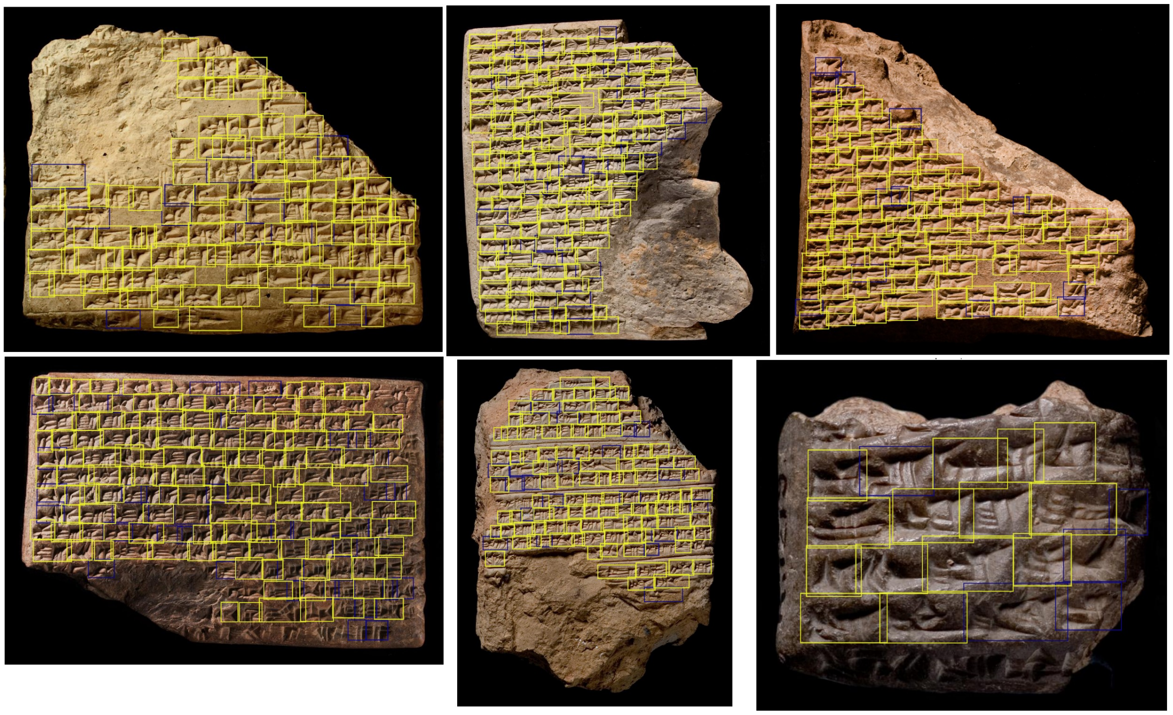

Figure H: Detection results on six tablet images from the test set with mostly clear cuneiform script. We only show detections with a confidence score higher than 0.5. Yellow bounding boxes indicate true positive detections and blue bounding boxes false positive detections. Best viewed in electronic form.

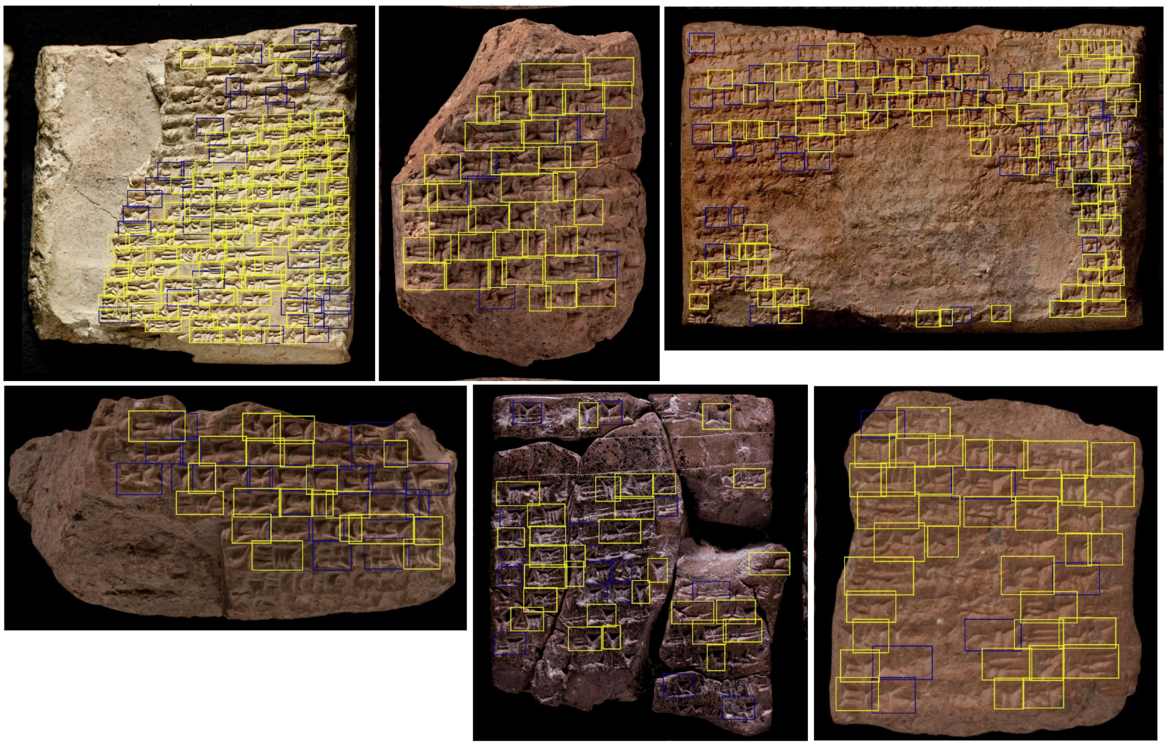

Figure I: Detection results on six tablet images from the test set with damaged cuneiform script. We only show detections with a confidence score higher than 0.5. Yellow bounding boxes indicate true positive detections and blue bounding boxes false positive detections. Best viewed in electronic form.

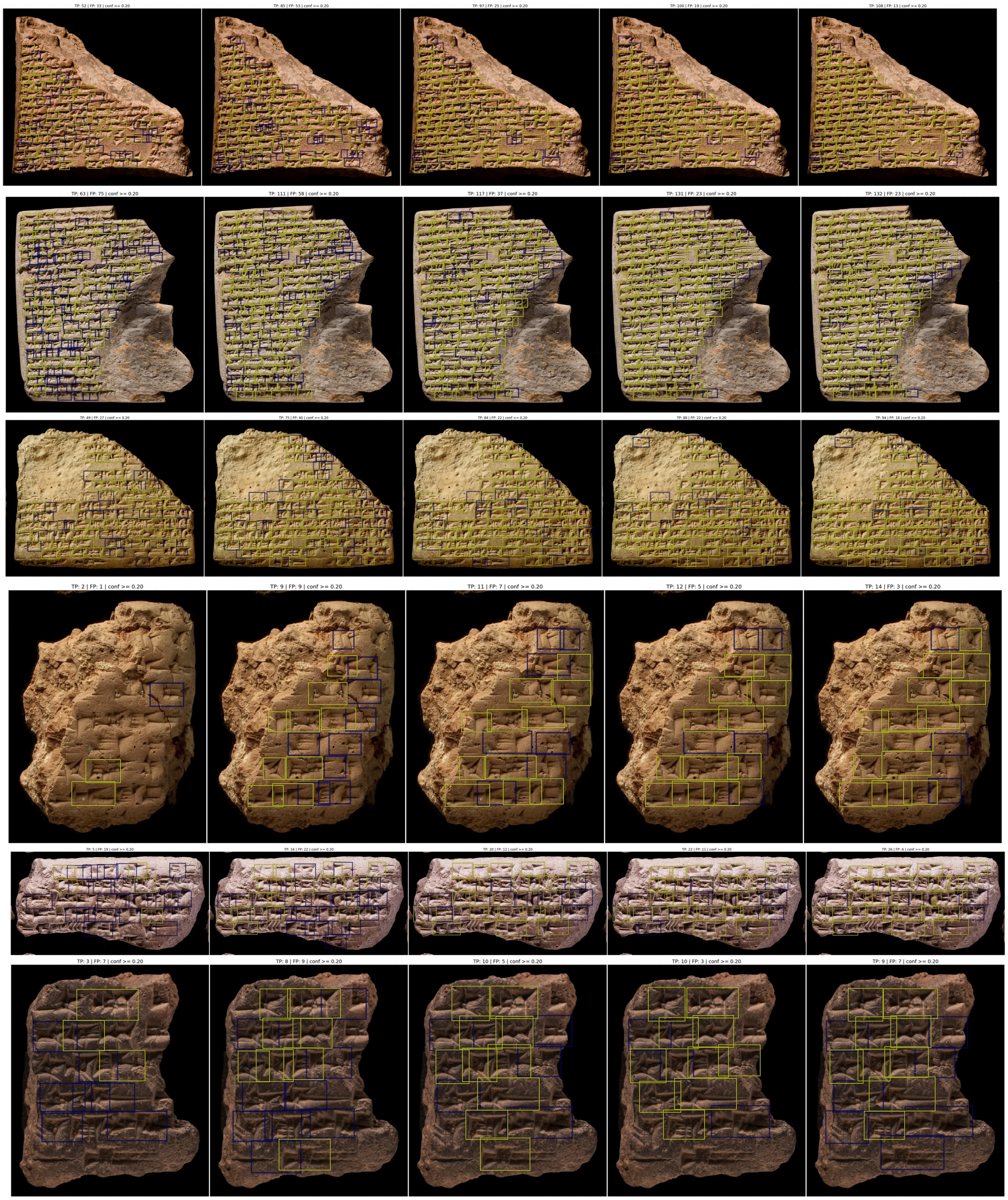

Figure J: Detection results on full tablet images for five consecutive iterations of iterative training (starting with first iteration from left): We only show detections with a confidence score higher than 0.2. Yellow bounding boxes indicate true positive detections and blue bounding boxes false positive detections. Best viewed in electronic form.

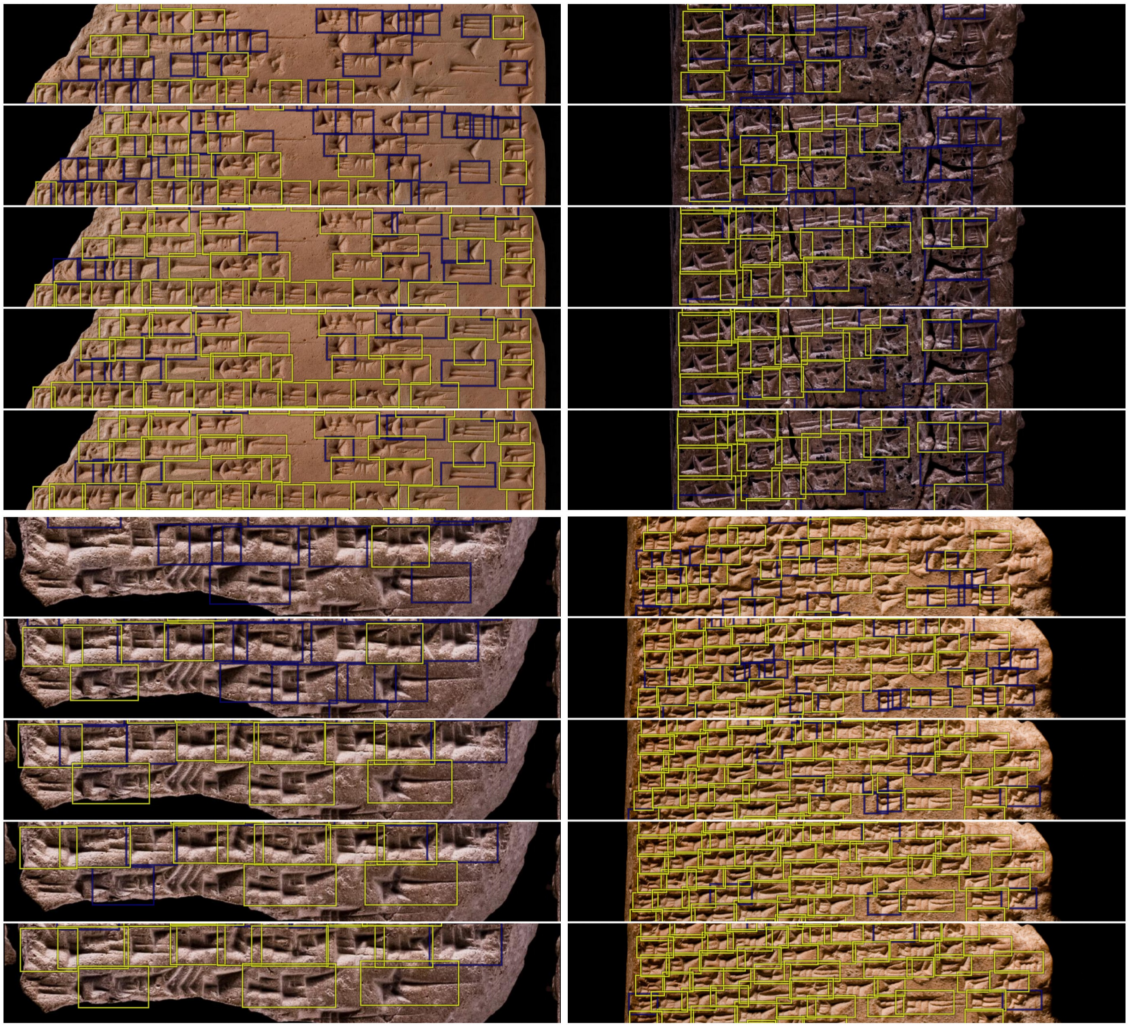

Figure K: Detection results on cropped image patches for five consecutive iterations of iterative training (starting with first iteration from top): We only show detections with a confidence score higher than 0.2. Yellow bounding boxes indicate true positive detections and blue bounding boxes false positive detections. Best viewed in electronic form.

architecture performs the worst, while the ResNet-18 model shows comparable performance to the *cuneiform* version, but requiring over ten times the number of parameters.

C6 ImageNet dataset pre-training

Pre-training a backbone network on the large ImageNet dataset [18] is a common method to improve the performance on different tasks with little training data. In Fig. L we investigate how well a backbone network performs on the task of cuneiform sign code classification when its training is initialized with ImageNet pre-trained weights compared to when it is trained from scratch. We use the same backbone architectures and training configuration as described in previous experiment. Initializing the network training with ImageNet pre-trained weights results for each architecture in a 1%–5% performance improvement when compared to training from scratch. This is in line with current research on transfer learning [19] that finds only modest performance improvements in the case of fine-grained target domain. In addition the domain of ImageNet objects is very different from the domain of cuneiform sign code classes and distinguishing them requires to learn two different feature representations with little overlap. Overall ImageNet pre-training offers a small performance improvement, however, it is clear that it cannot replace the sign annotations required for detector training.

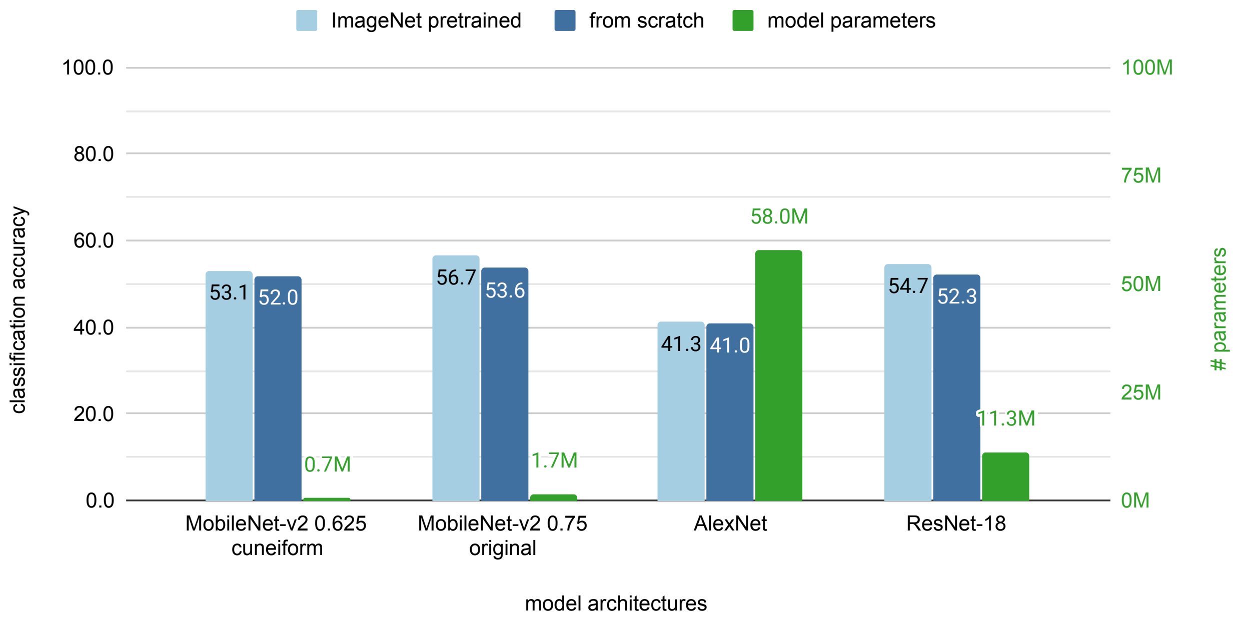

Figure L: Evaluation of different backbone architectures and their initialization on the task of cuneiform sign code classification. Four backbone architectures trained from scratch are compared to ones trained from ImageNet pre-trained weights. The performance is measured on the test set as classification accuracy (%).

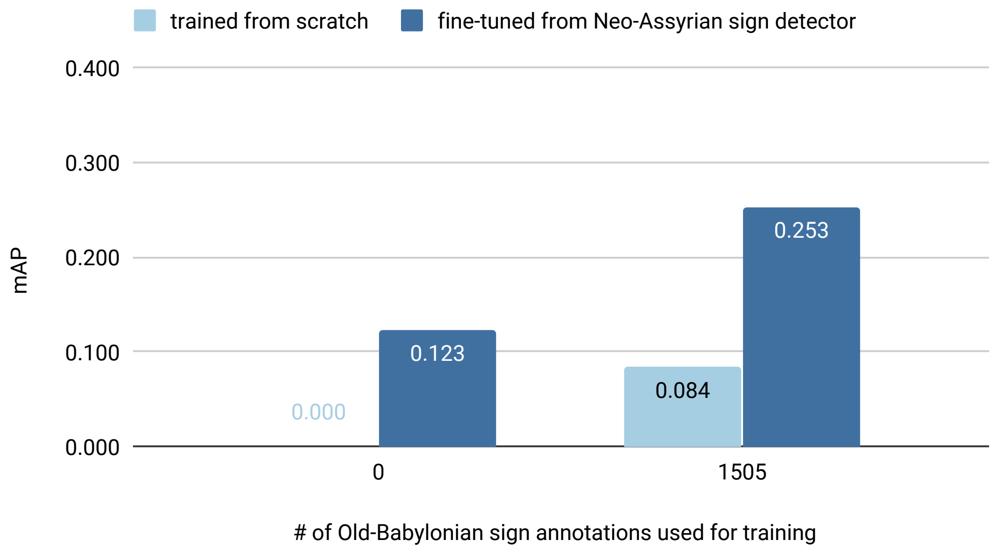

Figure M: Comparison of two ways to train a Old-Babylonian sign detector on 1505 bounding box annotations of Old-Babylonian cuneiform. A sign detector trained from scratch is compared to one fine-tuned from a Neo-Assyrian sign detector that has been pre-trained with our iterative learning procedure. The performance is measured on a test set made up of clay tablets from CUSAS 36 collection [20].

C7 Apply Sign Detector To Different Cuneifrom Script

We investigate if our approach is transferable to Old Babylonian cuneiform. Old-Babylonian shares most of the sign code classes of Neo-Assyrian cuneiform, however, the majority of sign code classes is written differently. For our experiment we annotated 18 Old-Babylonian clay tablets of the CUSAS 36 collection [20], of which twelve were used for training (1505 signs) and six for testing (591 signs). Unfortunately, the transliterations of the CUSAS 36 collection are not publicly available

online at the time of writing, preventing us from employing our weakly-supervised learning strategy. Nevertheless, we show that a sign detector for Old-Babylonian script can be trained successfully and that fine-tuning a Neo-Assyrian sign detector on Old-Babylonian significantly improves the performance. This demonstrates that our sign detector take advantage of the close relationship between different cuneiform scripts.

We make use of the same training protocol as established for Neo-Assyrian cuneiform. First we train the Neo-Assyrian detector from scratch using only the sign annotations available in the train set. Then we compare this performance with a sign detector that was first trained for Neo-Assyrian and then fine-tuned for Old-Babylonian cuneiform. To provide a lower bound we also apply a randomly initialized network and the Neo-Assyrian sign detector without any fine-tuning.

The results of the experiment is visualized in Fig. M. A Neo-Assyrian sign detector without any fine-tuning (12.9 mAP) already outperforms a purely supervised sign detector (8.4 mAP). The detection performance of 12.9 mAP can be interpreted as an estimate of the similarity between Neo-Assyrian and Old-Babylonian cuneiform, indicating some overlap between the two. When fine-tuning the Neo-Assyrian sign detector on Old-Babylonian cuneiform, we note a significant increase in performance (25.4 mAP). In order to close the gap to a Neo-Assyiran detector, more training data is required using manual sign annotations or levering the weak supervision in transliterations by means of our proposed approach.

C8 Web Application of Cuneiform Sign Detection

In the provided video, we demonstrate a web application of the cuneiform sign detector. The purpose of this demonstration is to illustrate how a sign detector could be made available to Assyriologists. Moreover, we provide the code of the web application. The web application offers the following core functionality: 1) create collections of tablet images, 2) upload tablet images, 3) apply the cuneiform sign detector, 4) visualize cuneiform sign detections, and 5) annotate cuneiform signs and lines. Please refer to the code repository for more details (link provided in main article).

In the video the following steps are shown: A tablet image is opened in the web interface, and the detection function is called. Since the image resolution of tablet images varies a lot, the user manually determines the average height of a line for re-scaling the tablet image to a standard sign height. A coarse approximation is sufficient, as the sign detector is searching across multiple scales by default. This step can be automated using line detection as implemented for the iterative training procedure. After running the detection, the results are immediately available for analysis in the web interface. Raw detections are visualized as bounding boxes, whose color indicates the detection confidence. The sign code class of the detected signs can be displayed in the form of readings or Unicode symbols (by pressing [.] or [ctrl] + [.] respectively) or by hovering with a cursor over the signs. It is possible to adjust thresholds to filter raw detections in two ways: 1) Raw detections can be filtered according to their confidence values, only displaying detections with a minimum confidence. 2) Raw detections can be filtered by means of non-maximum suppression (NMS): For each pair of raw detections compute the overlap of their bounding boxes and if two boxes have an overlap larger than the selected NMS-threshold remove the detection with the lower detection confidence.

Besides supporting the analysis of cuneiform tablets, the web interface can also facilitate the manual annotation process for semi-supervised training: Instead of annotating tablet images from scratch, an expert first applies the trained sign detector from our approach to obtain sign detections as a starting point, thus speeding up the annotation process significantly.

References

[1] Alex Krizhevsky, Ilya Sutskever, and Geoffrey E Hinton. Imagenet classification with deep convolutional neural networks. In *Advances in neural information processing systems*, pages 1097–1105, 2012.

[2] Richard O Duda and Peter E Hart. Use of the hough transformation to detect lines and curves in pictures. *Communications of the ACM*, 15(1):11–15, 1972.

[3] Sylvie Vanséveren. Unicode cuneiform fonts for macintosh and windows. https://www.hethport.uni-wuerzburg.de/cuneifont/, accessed May 20, 2018.

[4] Wei Liu, Dragomir Anguelov, Dumitru Erhan, Christian Szegedy, Scott Reed, Cheng-Yang Fu, and Alexander C Berg. Ssd: Single shot multibox detector. In *European conference on computer vision*, pages 21–37. Springer, 2016.

[5] Tsung-Yi Lin, Piotr Dollár, Ross Girshick, Kaiming He, Bharath Hariharan, and Serge Belongie. Feature pyramid networks for object detection. In *Proceedings of the IEEE conference on computer vision and pattern recognition*, pages 2117–2125, 2017.

[6] Rico Sennrich and Martin Volk. Iterative, mt-based sentence alignment of parallel texts. In *Proceedings of the 18th Nordic Conference of Computational Linguistics (NODALIDA 2011)*, pages 175–182, 2011.

[7] Kishore Papineni, Salim Roukos, Todd Ward, and Wei-Jing Zhu. Bleu: a method for automatic evaluation of machine translation. In *Proceedings of the 40th annual meeting on association for computational linguistics*, pages 311–318. Association for Computational Linguistics, 2002.

[8] Pedro F Felzenszwalb and Daniel P Huttenlocher. Pictorial structures for object recognition. *International journal of computer vision*, 61(1):55–79, 2005.

[9] Vladimir Kolmogorov. Convergent tree-reweighted message passing for energy minimization. *IEEE transactions on pattern analysis and machine intelligence*, 28(10):1568–1583, 2006.

[10] Bjoern Andres, Thorsten Beier, and Jörg H Kappes. Opengm: A c++ library for discrete graphical models. *arXiv preprint arXiv:1206.0111*, 2012.

[11] Ramprasaath R Selvaraju, Michael Cogswell, Abhishek Das, Ramakrishna Vedantam, Devi Parikh, and Dhruv Batra. Grad-cam: Visual explanations from deep networks via gradient-based localization. In *Proceedings of the IEEE International Conference on Computer Vision*, pages 618–626, 2017.

[12] Adam Paszke, Sam Gross, Francisco Massa, Adam Lerer, James Bradbury, Gregory Chanan, Trevor Killeen, Zeming Lin, Natalia Gimelshein, Luca Antiga, et al. Pytorch: An imperative style, high-performance deep learning library. In *Advances in neural information processing systems*, pages 8026–8037, 2019.

[13] Rykle Borger. *Mesopotamisches Zeichenlexikon*. Alter Orient und Altes Testament. Ugarit-Verl., Münster, 2004.

[14] Derek Hoiem, Yodsawalai Chodpathumwan, and Qieyun Dai. Diagnosing error in object detectors. In *Proceedings of the 12th European Conference on Computer Vision - Volume Part III*, ECCV'12, pages 340–353, Berlin, Heidelberg, 2012. Springer-Verlag.

[15] Jonathan Huang, Vivek Rathod, Chen Sun, Menglong Zhu, Anoop Korattikara, Alireza Fathi, Ian Fischer, Zbigniew Wojna, Yang Song, Sergio Guadarrama, et al. Speed/accuracy trade-offs for modern convolutional object detectors. In *Proceedings of the IEEE conference on computer vision and pattern recognition*, pages 7310–7311, 2017.

[16] Mark Sandler, Andrew Howard, Menglong Zhu, Andrey Zhmoginov, and Liang-Chieh Chen. Mobilenetv2: Inverted residuals and linear bottlenecks. In *Proceedings of the IEEE Conference on Computer Vision and Pattern Recognition*, pages 4510–4520, 2018.

[17] Kaiming He, Xiangyu Zhang, Shaoqing Ren, and Jian Sun. Deep residual learning for image recognition. In *Proceedings of the IEEE conference on computer vision and pattern recognition*, pages 770–778, 2016.

[18] Jia Deng, Wei Dong, Richard Socher, Li-Jia Li, Kai Li, and Li Fei-Fei. Imagenet: A large-scale hierarchical image database. In *2009 IEEE conference on computer vision and pattern recognition*, pages 248–255. Ieee, 2009.

[19] Simon Kornblith, Jonathon Shlens, and Quoc V Le. Do better imagenet models transfer better? In *Proceedings of the IEEE conference on computer vision and pattern recognition*, pages 2661–2671, 2019.

[20] Andrew George. *Old Babylonian Texts in the Schøyen Collection, Part One: Selected Letters. (Cornell University Studies in Assyriology and Sumerology 36)*. CDL Press, 2017.
